# Comparative genomics of the *Liberibacter* genus reveals widespread diversity in genomic content and positive selection history

**DOI:** 10.3389/fmicb.2023.1206094

**Published:** 2023-06-26

**Authors:** Tiffany N. Batarseh, Sarah N. Batarseh, Abraham Morales-Cruz, Brandon S. Gaut

**Affiliations:** ^1^Department of Integrative Biology, UC Berkeley, Berkeley, CA, United States; ^2^Department of Plant and Microbial Biology, UC Berkeley, Berkeley, CA, United States; ^3^U.S. Department of Energy, Joint Genome Institute, Lawrence Berkeley National Lab, Berkeley, CA, United States; ^4^Department of Ecology and Evolutionary Biology, UC Irvine, Irvine, CA, United States

**Keywords:** evolution, bacteria, pan-genome, positive selection, plant pathogen

## Abstract

‘*Candidatus* Liberibacter’ is a group of bacterial species that are obligate intracellular plant pathogens and cause Huanglongbing disease of citrus trees and Zebra Chip in potatoes. Here, we examined the extent of intra- and interspecific genetic diversity across the genus using comparative genomics. Our approach examined a wide set of *Liberibacter* genome sequences including five pathogenic species and one species not known to cause disease. By performing comparative genomics analyses, we sought to understand the evolutionary history of this genus and to identify genes or genome regions that may affect pathogenicity. With a set of 52 genomes, we performed comparative genomics, measured genome rearrangement, and completed statistical tests of positive selection. We explored markers of genetic diversity across the genus, such as average nucleotide identity across the whole genome. These analyses revealed the highest intraspecific diversity amongst the ‘*Ca.* Liberibacter solanacearum’ species, which also has the largest plant host range. We identified sets of core and accessory genes across the genus and within each species and measured the ratio of nonsynonymous to synonymous mutations (dN/dS) across genes. We identified ten genes with evidence of a history of positive selection in the *Liberibacter* genus, including genes in the Tad complex, which have been previously implicated as being highly divergent in the ‘*Ca.* L. capsica’ species based on high values of dN.

## 1. Introduction

Emerging and unmanaged bacterial plant pathogens threaten food security worldwide, with up to 10% of global food production lost to plant disease (Strange and Scott, 2005; McDonald and Stukenbrock, 2016; Ristaino et al., 2021). Understanding the mechanisms and genetic determinants that underlie diversification and adaptation in bacterial plant pathogens is essential for managing disease. The relationship between pathogen, diseased host, and associated vector creates arms-race dynamics that can fuel rapid evolutionary change in the pathogen (Anderson et al., 2010). The evolutionary consequences are reflected in the pathogen’s genome architecture, gene content, and patterns of genetic variation (Stavrinides et al., 2008). One such pattern is positive selection, an evolutionary force responsible for rapid adaptive change in pathogenic bacteria. Positive selection can be identified by measuring the ratio of nonsynonymous to synonymous mutations in genes, a ratio referred to as dN/dS or ω (Aguileta et al., 2009; Moulana et al., 2020). In the case of intracellular bacterial pathogens, evolutionary arms-race dynamics have also selected for genome rearrangements, reduced genome size, and a reduction in GC content, all of which have direct effects on phenotype and are correlated with pathogenicity (Murray C. S. et al., 2021). By investigating the genomes of plant pathogens for signatures of arms-race dynamics, we may identify genes or gene regions that are important for disease and also characterize the evolutionary processes underlying pathogen adaptation and evolution.

The *Liberibacter* genus is a group of gram-negative, alpha-Proteobacteria with species members that cause devastating disease on important crops around the world (Jagoueix et al., 1994). The genus comprises at least nine species: ‘*Candidatus* Liberibacter asiaticus’ (Las), ‘*Ca.* Liberibacter americanus’ (Lam), ‘*Ca.* Liberibacter africanus’ (Laf), ‘*Ca.* Liberibacter solanacearum’ (Lso), ‘*Ca.* Liberibacter europaeus’ (Leu), ‘*Ca.* Liberibacter brunswickensis’ (Lbr), ‘*Ca.* Liberibacter capsica,’ *‘Ca.* Liberibacter ctenarytainae,’ and *L. crescens* (Teixeira et al., 2005; Bové, 2006; Liefting et al., 2009; Raddadi et al., 2011; Morris et al., 2017; Jain et al., 2019; Kwak et al., 2021), each with varying pathogenic characteristics. The bacteria are largely phloem-restricted and vectored by psyllid insects.

Species from the *Liberibacter* genus cause a variety of plant diseases. For example, Las, Lam, and Laf - which are predominantly found in Asia, the Americas, and Africa, respectively - cause Huanglongbing (HLB) disease. HLB is a destructive disease of citrus (plant family *Rutaceae*) that causes multibillion-dollar economic loss worldwide per year (Bové, 2006; Yuan et al., 2021). The disease in citrus is characterized by yellowing of leaves, reduced fruit yield, and twig dieback, which results in non-marketable fruit leading to great commercial losses (Albrecht and Bowman, 2009; Bassanezi et al., 2011). HLB can be carried and spread by insect psyllids, such as *Trioza erytreae* in Africa and *Diaphorina citri* in America and Asia, or it can be spread by grafting (Reynaud et al., 2022). Although all three species can cause HLB, Las is most frequently identified in positive samples, and it has even outcompeted Lam in its native Brazil (Coletta-Filho et al., 2010). Preventing HLB is a global concern as there are no widely implemented and sustainable cures available (Alquézar et al., 2021).

Outside of citrus, other *Liberibacter* species colonize and infect different plant hosts. Lso is known to infect solanaceous plants (*Solanaceae*) and plants in the family *Apiaceae*. The identification of Lso marked the first report of *Liberibacter* naturally infecting a host byond citrus, which is in the *Rutaceae* (Liefting et al., 2009). Lso is the causal agent of Zebra Chip (ZC) disease in potatoes, which is characterized by brown stripes in the potato tuber due to infection of the phloem and causes major yield losses (Mora et al., 2021). Lso has also been discovered to infect carrots in Finland, New Zealand, and North and Central America (Munyaneza et al., 2010), suggesting that Lso infects a greater diversity of plant hosts compared to the other species. Leu was first discovered through a screen for *Liberibacter* species in *Cacopsylla pryi,* a psyllid pear pest in Italy, though it did not confer disease on pear plant tissue (Raddadi et al., 2011). However, Leu has been implicated as the causal agent behind disease symptoms in Scotch Broom shrubs (*Cytisus scoparius*) in New Zealand and associated with the broom psyllid, *Arytainilla spartiophila,* suggesting that Leu has more than one cognate host-psyllid pair and causes variable symptoms based on the host (Thompson et al., 2013).

Understanding the genetic determinants behind devastating diseases is imperative because it can lead to early detection and effective management strategies. Unfortunately, the majority of described species of *Liberibacter* are unable to be reliably cultured and propagated. However, *L. crescens* is a culturable species and is ancestral to all other described ‘*Ca.* Liberibacter’ species (Jain et al., 2019; Merfa et al., 2022). Due to their fastidious nature, obtaining genomic sequences of ‘*Ca. Liberibacter*’ species typically requires metagenomic approaches on infected plant tissue or insect vectors. Because of this obstacle, genetic manipulation of ‘*Ca.* Liberibacter’ remains difficult and hinders complete understanding of their pathogenicity mechanics and the underlying genetic determinants (Levy et al., 2020). Despite this obstacle, recent experimental work has shown that Las induces a systemic and chronic immune response in the phloem tissue in *Citrus sinensis,* thus demonstrating its pathogenicity (Ma et al., 2022).

In this work, we take the view that genomic analyses among *Liberibacter* species may help identify genetic determinants important for pathogenicity and improve our understanding of genomic variation in the genus. Here, we compare genomic diversity among six species of the *Liberibacter* genus using publicly available and assembled genome sequences. We are not the first to compare the genome content in *Liberibacter* species (Thapa et al., 2020; Gao et al., 2022). For example, Thapa et al. (2020) recently studied a sample of 37 *Liberibacter* genomes to investigate systematic relationships and genetic diversity within species. Others have focused on the evolution of prophage sequences within *Liberibacter* genomes (Dominguez-Mirazo et al., 2019; Tan et al., 2021). Previous studies have provided evidence for inter- and intraspecies genomic rearrangements, but without characterizing the extent of structural variation within and between species. Our work differs from previous studies by analyzing more genomes and also by focusing on the evolutionary history of gene presence/absence - i.e., the ‘accessory’ genome. Our work also differs by applying dN/dS analyses to the entire gene set, including accessory genes, as a method to identify candidate genes that may function in arms-race dynamics via host-pathogen interactions. Altogether, our work compiles a dataset of >50 *Liberibacter* genomes, compares nucleotide identity among species, performs pan-genome analyses to investigate gene content variation, measures gene presence/absence, and applies statistical analyses of dN/dS to both core and accessory genes. These analyses provide unprecedented insights into the evolutionary dynamics and history of ‘*Candidatus* Liberibacter’ genomes.

## 2. Methods

### 2.1. *Liberibacter* whole genome sequence sample selection

We obtained all publicly available genomes of the genus *Liberibacter* from the National Center for Biotechnology Information (NCBI) and Pathosystems Resource Integration Center (PATRIC) database on March 24th, 2022. We obtained 36 genomes of ‘*Ca.* Liberibacter asiaticus,’ 2 genomes of ‘*Ca.* Liberibacter americanus,’ 2 genomes of ‘*Ca.* Liberibacter europaeus,’ 2 genomes of ‘*Ca.* Liberibacter africanus,’ and 8 genomes of ‘*Ca.* Liberibacter solanacearum’ (Supplementary Table S1). Additionally, we included 2 genomes of *Liberibacter crescens* (Table 1). Genome completeness was assessed using CheckM v 1.2.0 (Parks et al., 2015), based on a set of conserved single copy genes (Supplementary Table S2). Three genomes of ‘*Ca.* Liberibacter asiaticus’ were excluded (genomes Tabriz3, SGCA_1, and SGpsy) because their genome sizes were considerably lower than the accepted 1.2 Mb genome size of Las (0.769 Mb and 0.23 Mb) or their genome completeness was less than 80% based on CheckM, likely reflecting incomplete genomes.

**Table 1:** Summary of *Liberibacter* accessions included in this study.

Species	Culturable	Abbreviation	Number of accessions	Plant hosts (known disease)	References
*Liberibacter crescens*	Yes	*L. crescens*	2	N/A	Leonard et al. (2012)
‘*Ca. Liberibacter* asiaticus’	No	Las	36	*Rutaceae*	Jagoueix et al. (1994)
‘*Ca. Liberibacter* solanacearum’	No	Lso	8	*Solanaceaous* and *Apiaceous*	Munyaneza et al. (2009), Munyaneza et al. (2010), and Hajri et al. (2017)
‘*Ca. Liberibacter* africanus’	No	Laf	2	*Rutaceae*	Roberts et al. (2015)
‘*Ca. Liberibacter* americanus’	No	Lam	2	*Rutaceae*	Teixeira et al. (2005)
‘*Ca. Liberibacter* europaeus’	No	Leu	2	*Cytisus scoparius* (*Fabaceae*)	Tannières et al. (2020)

### 2.2. Pan-genome analysis and gene annotation

All genomes retrieved from public databases were annotated using Prokka v 1.14.6 in order to obtain uniform annotations (Seemann, 2014). Following annotation, a genus-wide pan-genome was constructed using Roary v. 3.13.0 with *Rhizobia grahamii* as the outgroup (Table 2). Due to the high divergence amongst *Liberibacter* species, we decreased the percentage sequence identity to 50% to encompass all possible orthologs following previously established methods (Chase et al., 2018; Rodriguez and Martiny, 2020). Using the pan genome reference fasta file output by Roary, we extracted a representative nucleotide sequence of each core and accessory gene using cdbfasta.^1^ Theses gene sequences were translated using the transeq command from EMBL-EBI (Madeira et al., 2019). Functional categorization of the core genes and accessory genes was performed using the program eggNOG-mapper v. 2 (Huerta-Cepas et al., 2017, 2019). KEGG annotation was performed using the GhostKoala online server (Kanehisa et al., 2016). Additionally, we measured the pairwise average nucleotide identity (ANI) between all genomes used in this study using OrthoANI v. 1.2 (Lee et al., 2016).

**Table 2:** Pan-genome analysis.

Pan-genome Analysis	Number of genomes	Number of core genes	Number of accessory genes^1^	Total genes excluding singletons	Total genes
Genus wide with outgroup	53	436	2,639	3,075	8,926
Genus wide without outgroup	52	496	2,619	3,115	3,982
*L. crescens*	2	1,291	89	–	1,380
Laf	2	976	229	–	1,205
Lam	2	936	65	–	1,001
Las	36	786	768	1,554	2,015
Leu	2	1,100	44	–	1,144
Lso	8	822	547	1,369	1,900

1 Excluded singletons for all analyses with >2 genomes.

### 2.3. Phylogenetic tree construction

The core gene alignment was used to build a phylogenetic tree representing the entire genus. The core gene alignment produced by Roary was polished using the gBlocks program (Castresana, 2000). The polished core gene alignment was inputted to RAxML v. 8.2.12 to build a phylogenetic tree (Stamatakis, 2014). In order to further evaluate and curate the isolates, we created a distance matrix from the RAxML phylogenetic tree in newick format using the Tree and reticulogram REConstruction (T-REX) server (Boc et al., 2012). Visualization of the phylogenetic tree built from core gene sequences was performed using the Interactive Tree of Life (iTOL; Letunic and Bork, 2021). Additionally we explored the phylogenetic relatedness of the accessions based on accessory gene content. We constructed a distance matrix based on the presence-absence patterns of accessory genes and then used that matrix to build a neighbor-joining tree. To build the tree, we calculated the Euclidean distances from the presence-absence matrix using the *dist* function in R (R Core Team, 2021). The distance matrix based on accessory gene content was then used as input to build the tree using the ape package in R (Paradis et al., 2004). Using the Analysis of Similarities (ANOSIM) in the R *vegan* package, we tested the association between the accessory gene content and species identity (Oksanen et al., 2020).

### 2.4. Whole genome alignment and genome rearrangement

To investigate the rearrangement history of *Liberibacter* and to generate a multiple whole genome alignment, we used the progressiveMauve system available through Mauve (Darling et al., 2004). Before performing multiple genome alignment with Mauve, we used the Mauve Contig Mover (MCM) on assemblies with >1 contig using the Las isolate Psy62 or Lso isolate CLsoZC1 as the reference genome, depending on the analysis (Darling et al., 2007). ProgressiveMauve was run with default settings and the Locally Collinear Blocks (LCBs) that were found across all genomes were extracted and concatenated into a multiple genome alignment. We performed progressiveMauve analysis with 10 different sets of genomes: a set of only single contig assemblies (*n* = 16), a set of genomes comprising two representative sequences per *Liberibacter* species (*n* = 12), and 8 different species-specific analyses (Table 3). For the interspecific analyses, Psy62 was used as the reference genome for MCM. However, for the Lso species-specific analyses, we used CLsoZC1 as the reference genome for MCM. For the species-specific analysis of Las genomes, we used subsets of Las genomes with *n* = 10 or *n* = 8 because Mauve was too computationally intensive to run the analyses on all 36 Las genomes. We therefore investigated three sampling subsets. The first subset was composed of all 10 single-contig Las assemblies (chromosome level); the second was composed of a random subsample of 8 Las genomes from the entire set of 36 genomes; and the third set included a random subsample of 8 Las genomes from the set of 26 Las genomes composed of >1 contig. We selected 8 Las genomes for the second and third analysis sets in order to match the number of available Lso genomes (*n* = 8).

**Table 3:** *Liberibacter* progressiveMauve analysis.

Mauve analysis set	Number of genomes	Number of species	LCBs	Nucleotides	Percent identity
Chromosome assemblies only	16	5	136	1,167,307	60.5
All species representation	12	6	156	1,148,204	44.6
Las (chromosome assemblies only)	10	1	3	1,200,837	97.9
Las (random set of 8)	8	1	1	1,178,619	96.4
Las (random set of 8 excluding chromosome assemblies)	8	1	11	1,199,677	95.4
Lso	8	1	46	1,161,137	89
Leu	2	1	1	1,330,452	99.8
Laf	2	1	4	1,183,278	99.1
Lam	2	1	22	1,165,540	99.98
*L. crescens*	2	1	2	1,511,419	98.4

### 2.5. Measuring selection

We utilized the codeml set of models from PAML v.4.9 to calculate the ratio of nonsynonymous to synonymous substitution rates, known as dN/dS or ω, on the sets of single-copy core and single-copy accessory genes (Yang, 1997, 2007). For all models tested, we only considered genes with a minimum of four sequences (i.e., four different accessions carry the gene), as this is the minimum number recommended for codeml analysis.^2^ The codeml analysis in PAML requires the input of an unrooted, maximum-likelihood phylogenetic tree for each sequence analyzed, which we constructed using RAxML v. 8.2.12 using the gene sequence alignment as input for tree construction (Stamatakis, 2014).

We performed the codeml analysis under various models and compared the model results using likelihood ratio tests (Yang, 2007). Using the various models, we could test the null hypothesis that ω = 1.0 against the alternative of positive selection (ω > 1) using two different methods. First, in the global test approach, we calculated a single estimate of ω for each gene across its entire phylogeny. To test for the validity of the estimated value of ω, we performed a comparison of two models using the likelihood ratio test: one that estimated a single ω for each gene from the data (Model = 0 with Fix_omega = 0 in the codeml control file) and another model that calculated the likelihood if ω = 1.0 (Model = 0 with Fix_omega = 1 and Omega = 1 in the codeml control file). Using this set of models, we could identify genes with evidence of positive selection when the initial ω estimate was >1.0 and when the likelihood of the two models differed significantly based on *p* < 0.01 after FDR *p*-value correction. The second analysis consisted of the Sites models approach which tests for genes with variable selection pressure across sites, or codons, of the sequence. For each gene, we first compared Sites models M0 and M3 using the likelihood ratios to test for the presence of heterogeneity in evolutionary rates across codons. Any gene with a significant result for the M0–M3 comparison was then compared using Sites models M1a and M2a to identify specific codons with evidence of positive selection (ω > 1.0). For all summary statistics of ω, we excluded estimates of ω that were greater than 20 as potentially unreliable due either to low dS or poorly resolved optimization.

### 2.6. Statistical validation

All statistical analyses were performed in R v 4.1.2 (R Core Team, 2021). In order to compare the abundance of genes involved in KEGG functional pathways and ANI across species, we first assessed the normality of the data using the Shapiro–Wilk test and the variance of the data using Levene’s test available through R. For all comparisons of KEGG functional pathways, we performed ANOVA analyses using the R function *aov* and identified which groups were significantly different from each other by computing a *post hoc* Tukey HSD test with the R function *TukeyHSD*. For ANI comparisons, the Shapiro–Wilk test for normality was significant, so we proceeded with a Kruskal-Wallis analysis using the *kruskal.test* function in R. The Kruskal-Wallis test revealed significant differences between the groups, so we followed with the Pairwise Wilcoxon Rank Sum Test using the R function *pairwise.wilcox.test* with Benjamini and Hochberg p-value correction for multiple testing.

## 3. Results

### 3.1. Genus wide pan-genome analysis and phylogenetic relationships

To investigate the genus wide diversity and evolution of *Liberibacter* species, we obtained 52 *Liberibacter* whole genome sequences from public databases, representing one culturable and five nonculturable *Liberibacter* species. We also retrieved the genome sequence from *Rhizobium grahamii* as an outgroup sequence. Our complete sample of 53 whole genome sequences encompassed six *Liberibacter* species, with one species represented by 36 accessions (Table 1; Supplementary Table S1). We constructed the genus-wide pan-genome and categorized each gene as either a core or accessory gene in order to construct a core gene alignment to build a phylogeny. We defined a core gene as a gene that was present in a majority (95%) of the samples being tested (Tettelin et al., 2008). Across the entire genus, we identified 439 core and 8,487 accessory genes. Among accessory genes, 5,807 were singletons, meaning they were only identified in a single accession. We excluded singletons from further analysis and also excluded genes with an average number of sequences per isolate greater than one (due to splitting errors). Our final dataset consisted of 436 single-copy core genes and 2,639 single-copy accessory genes for downstream analysis (Table 2).

**Figure 1:**
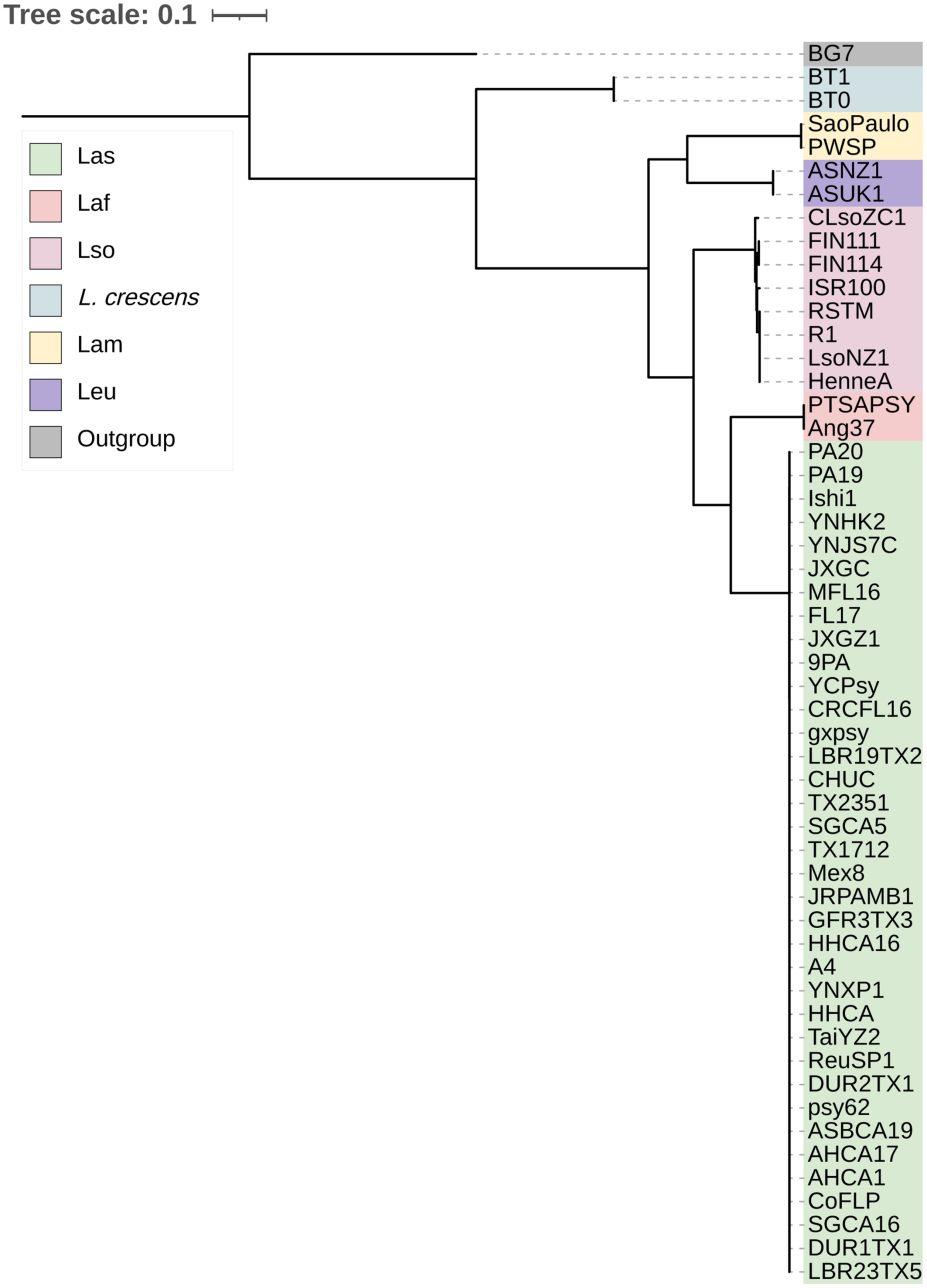
The maximum likelihood phylogeny of the 52 *Liberibacter* accessions and 1 outgroup species included in this study. The phylogeny was constructed using the nucleotide core gene alignment as the input. Each isolate is labeled at the tips and is colored according to the species designation.

With the genus wide pan-genome information, we constructed a core gene alignment to use as input to build a maximum likelihood phylogeny (Figure 1). As expected, the sequences clustered by species, with *L. crescens* basal among *Liberibacter* species (Wang N. et al., 2017). Following the split of *L. crescens* from the rest of the *Liberibacter* species, Lam and Leu sequences formed a clade that diverged from Lso, Laf, and Las. Overall, the phylogeny’s topology was highly supported with 100% bootstrap support across all nodes leading to species groups. Across all nodes in the tree, including within species clades, the median bootstrap support was 61.0% and the mean was 58.7% (Supplementary Figure S1). The lowest bootstrap supports were primarily found at nodes near the tips of the tree and only within species groups, such as in the Las clade, suggesting lower evolutionary divergence among these samples and their core gene sequences. The species tree based on core genes recapitulates the previous analysis of Thapa et al. (2020).

**Figure 2:**
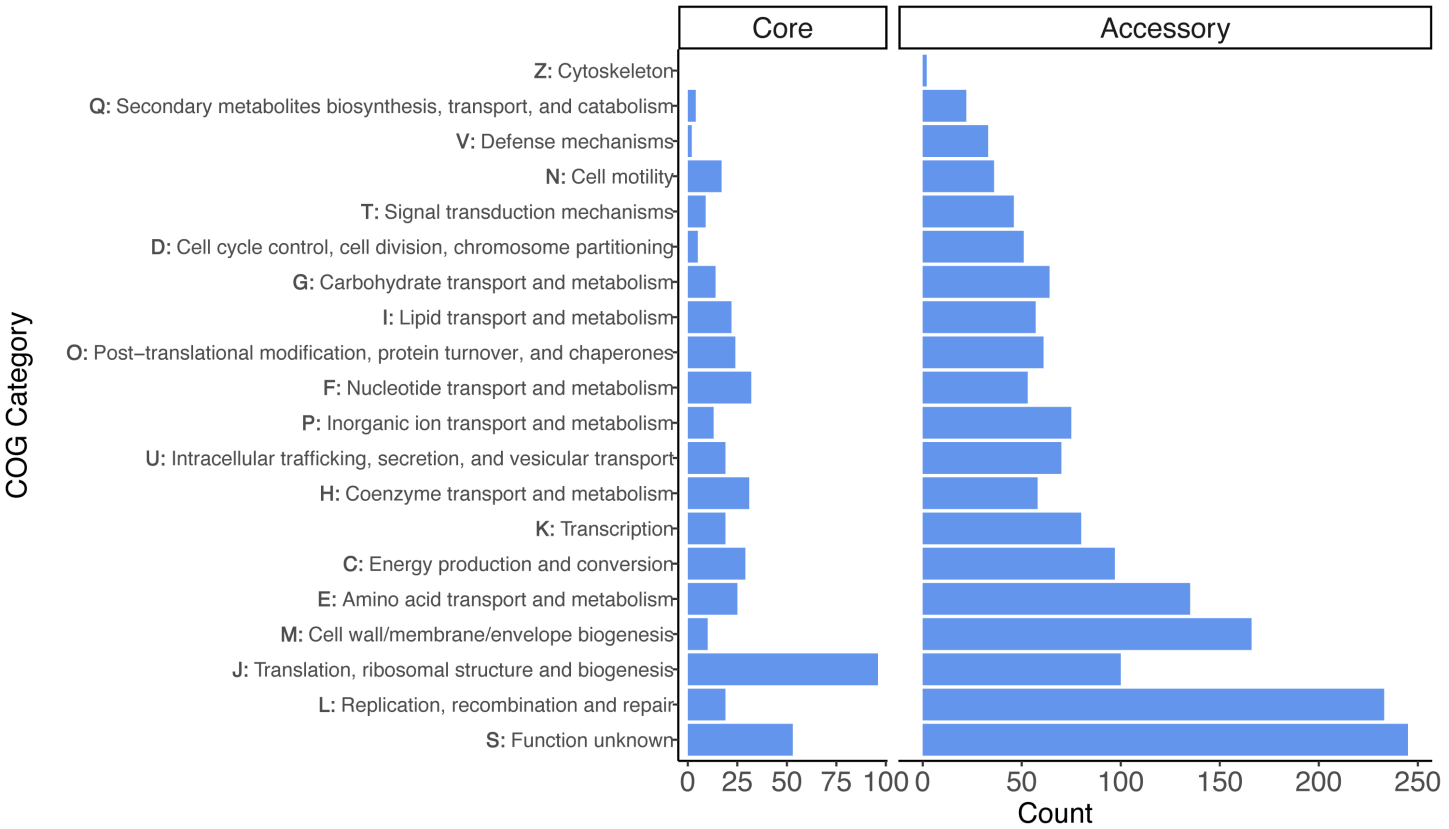
Gene annotation for the set of core and accessory genes identified across the *Liberibacter* genus. The distribution of functional categories are faceted for the set of 436 core genes and 2,639 accessory genes. The y-axis denotes both the COG category letter designation and the category description.

We performed functional analyses on the core and accessory gene sets using the COG annotation scheme (Figure 2). After excluding the category of S - ‘function unknown’ (which was the second largest category), the largest COG category among core genes was J - ‘translation, ribosomal structure, and biogenesis’ (96 genes), followed by F - ‘nucleotide transport and metabolism’ (32 genes) and H - ‘coenzyme transport and metabolism’ (31 genes). The accessory genes differed, because the largest categories (excluding ‘function unknown’ genes, which was the largest category) were L - ‘replication, recombination, and repair’ (233 genes), M - ‘cell wall/membrane/envelope biogenesis’ (166 genes), and E - ‘amino acid transport and metabolism’ (135 genes).

### 3.2. Nucleotide diversity across the genus

**Figure 3:**
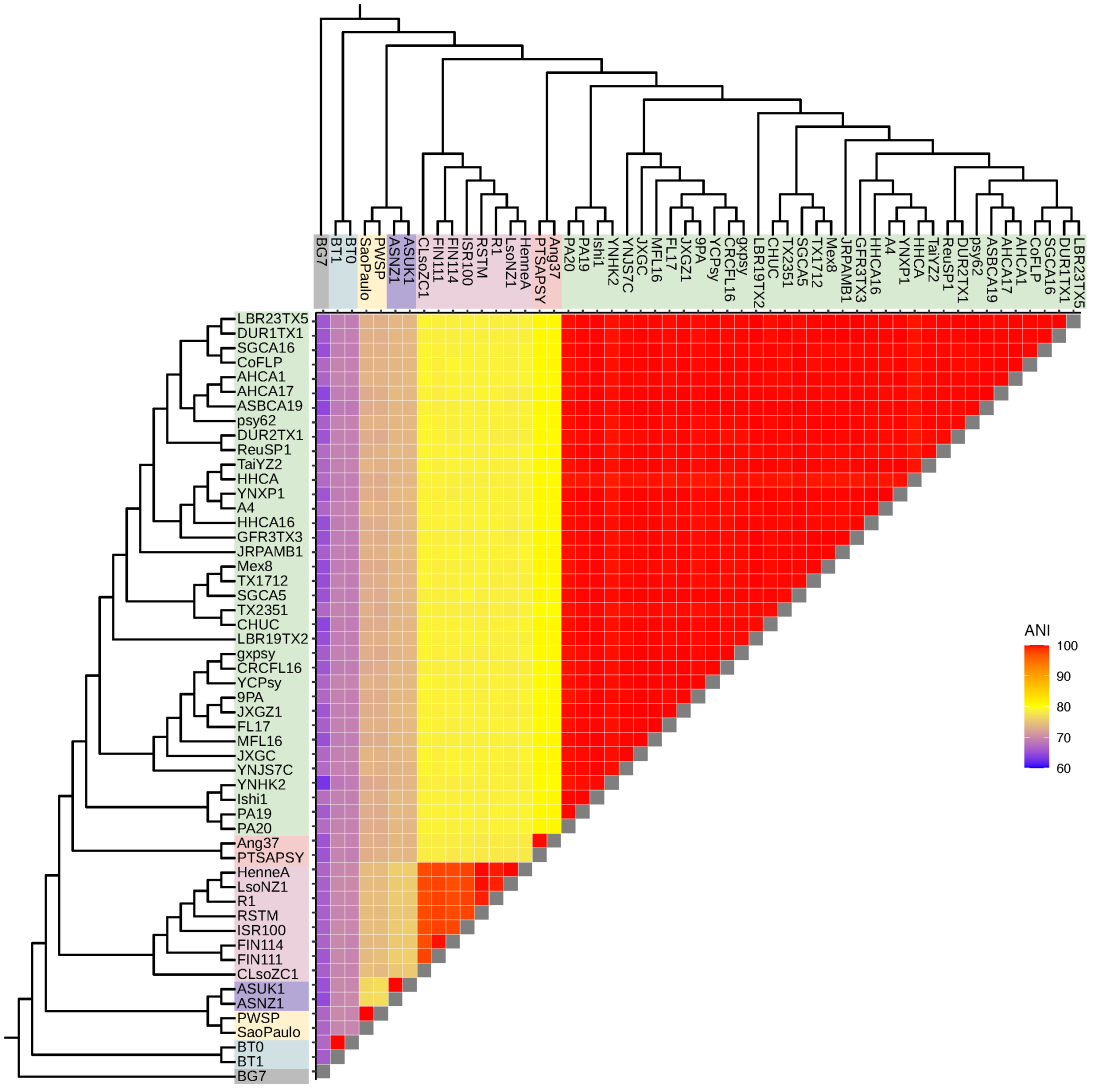
*Liberibacter* genome relatedness based on average nucleotide identity (ANI). ANI, measured in percent identity, was calculated between all possible pairs of accessions, resulting in 1,378 pairwise ANI calculations. The dendrograms represent the *Liberibacter* phylogeny based on the core gene alignment.

To investigate *Liberibacter* intra- and interspecies divergence, we calculated the genome-wide average nucleotide identity (ANI) using OrthoANI between all possible pairs of accessions included in this study, resulting in 1,378 pairwise ANI calculations (Figure 3). ANI is a commonly used metric to demarcate species and genus boundaries in bacteria (Jain et al., 2018). OrthoANI calculates high resolution ANI across the entire genome by fragmenting the genome sequences into 1,020 bp long fragments that are used for analysis (Lee et al., 2016). Across all accessions (including the outgroup species), the mean ANI value was 87.13% with a minimum value of 62.85% and a maximum of 99.99%. As expected, comparisons performed within species had a significantly higher ANI compared to the ANI calculated from across species comparisons (*p* < 2.2 × 10^−16^, Wilcox Test). Specifically, average ANI for between species comparisons was 76.2% (excluding calculations against the outgroup) while average ANI within species was 99.8%. As expected, ANI both within and between *Liberibacter* species were significantly different to the ANI calculated against the outgroup species (mean ANI against outgroup = 66%, *p* < 2.2 × 10^−16^, Kruskal–Wallis test; Figure 3).

Next, we investigated ANI within the *Liberibacter* species. We hypothesized that Lso isolates may have greater nucleotide diversity, and therefore lower mean ANI, compared to other *Liberibacter* species, like Las, which in turn may underlie the broader host range of Lso. We focused on the within-Lso and within-Las ANI comparisons as these two species had the greatest number of available genomes with 8 and 36 genomes, respectively. The mean within-Las ANI was 99.89% while the mean within-Lso ANI was 98.17% (Supplementary Figure S2). ANI was significantly higher for within-Las comparisons than within-Lso comparisons, indicating that Lso has greater nucleotide diversity compared to Las genomes (*p* < 0.0001, Wilcox Test). All other within-species calculations for Laf, Lam, Leu, and *L. crescens* resulted in ANI values of >99%, but only two genomes were available for each of these species. Overall, however, these analyses suggest that Lso has greater within-species nucleotide diversity compared to other *Liberibacter* species.

### 3.3. Species specific genome differences

We also investigated genomic differences among species. There were significant differences in total genome size between species (*p* = 0.001, Kruskal–Wallis test); for example Las isolates tended to have significantly smaller genomes compared to *L. crescens* and Leu isolates (*p* < 0.05). We also identified species’ differences by counting the core and accessory genes for each species individually (Table 2; Supplementary Figure S3). Intraspecies pan-genome analysis returned an average of 985 core genes per species with a minimum of 786 core genes in Las and a maximum of 1,291 core genes in *L. crescens*. A total of 536 genes were shared among the pathogenic species (Supplementary Figure S3A). The accessory gene count was more variable among the species, with an average of 291 accessory genes per species but a minimum and maximum of 44 (in Leu) and 768 (in Las). We recognize that the number of accessory genes is positively correlated with the number of genomes analyzed up to a saturation point (Bosi et al., 2015); hence, we are undoubtedly underestimating the number of accessory genes in species with only two genomes available. Among species with only two genomes available, Laf had 229 accessory genes, Lam had 65, and Leu had 44 resulting in an average of 112 accessory genes in these species.

**Figure 4:**
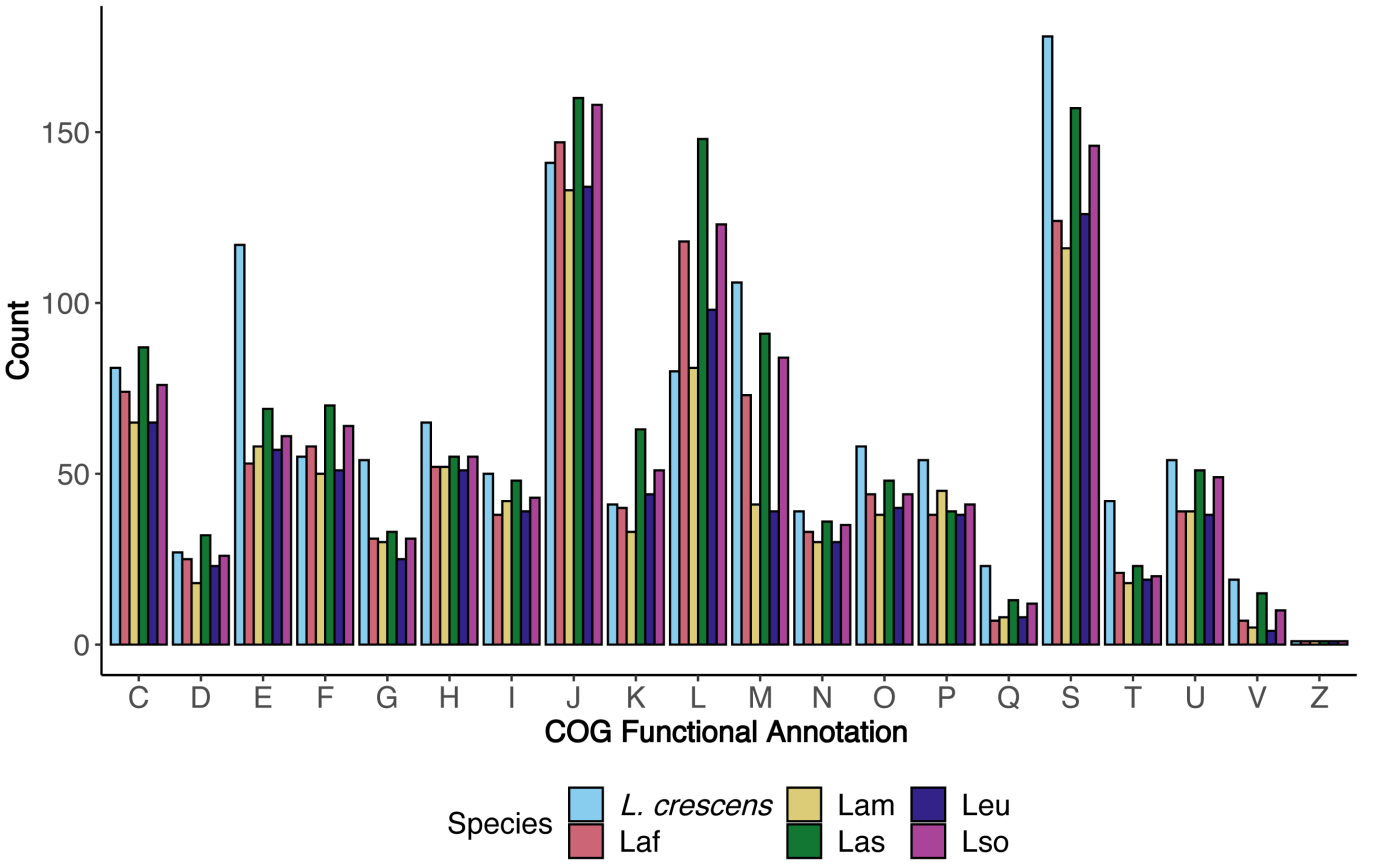
The distribution of functional categories for core and accessory genes separated by species. For each COG functional category, the number of genes annotated with that particular COG function in each species are depicted and separated by color. The COG definitions per letter are the same as in Figure 2.

Using the COG functional annotation scheme, we explored the species-specific core and accessory gene functions together (Figure 4). As expected (Merfa et al., 2019), *L. crescens* tended to have more genes with annotations in particular COG functional categories such as E - ‘amino acid transport and metabolism,’ H - ‘coenzyme transport and metabolism,’ O - ‘post-translational modification, protein turnover, and chaperones,’ P - ‘inorganic ion transport and metabolism,’ Q - ‘secondary metabolites biosynthesis, transport, and catabolism,’ and T - ‘signal transduction mechanisms,’ which may contribute towards *L. crescens* ability to be cultured or may be due to an inherently larger chromosome. Excluding *L. crescens*, the distribution amongst the COG categories was fairly consistent among the unculturable ‘*Ca.* Liberibacter’ species. COG categories J, L, and M revealed interesting patterns amongst Las, Laf, and Lso, as all three species tended to have higher counts in these categories compared to Lam and Leu. These categories were ‘translation, ribosomal structure, and biogenesis,’ ‘replication, recombination, and repair,’ and ‘cell wall/membrane/envelope biogenesis,’ respectively.

We compared and contrasted the core and accessory gene content between Las and Lso isolates, as these two species had >2 genomes available, allowing for greater confidence in the core and accessory gene representation in these species. Las and Lso had a total of 786 and 822 core and 768 and 547 accessory genes, respectively. In regard to core genes, the majority (75%) of core genes were the same between the two species as they shared 689 core genes in common. Each species had a set of unique core genes, however, with Las having 97 and Lso having 133 unique core genes. The accessory genes proved to differentiate the two species further, as Las and Lso only shared 354 accessory genes which represented just 37% of the total genes. The Lso species harbored a set of 414 unique accessory genes that were not a part of the Las accessory gene set, while Las only had a set of 193 unique accessory genes. Overall, this comparison suggests that Lso not only harbors more genetic diversity within core genes, as measured by ANI, but also more variation in accessory gene content.

### 3.4. Differences in abundance of genes involved in KEGG functional pathways

**Figure 5:**
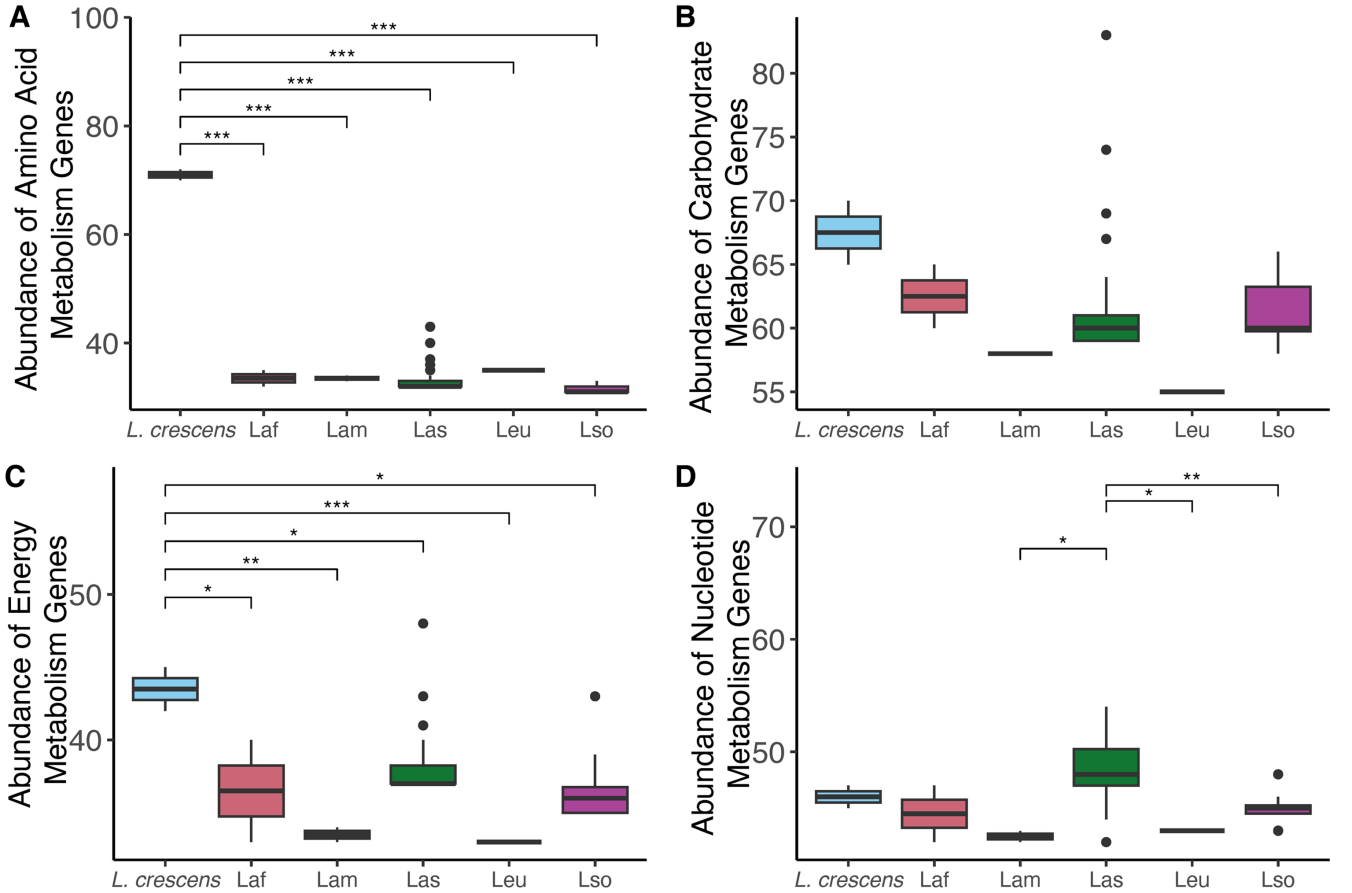
The abundances of genes involved in four different KEGG metabolism pathways amongst the different *Liberibacter* species: **(A)** amino acid, **(B)** carbohydrate, **(C)** energy, and **(D)** nucleotide. Abundance refers to the total gene count, and each boxplot represents the total number of genes annotated for that KEGG function in each genome analyzed in this study. Significance was assessed using ANOVA with *post hoc* Tukey HSD test, and the stars note the level of significance such that *p < 0.5, **p < 0.01, ***p < 0.001.

Since we found differences in gene content among the *Liberibacter* species, we compared and contrasted the abundance of genes involved in different processes essential to the biology of the cell: nucleotide, amino acid, energy, and carbohydrate metabolism using KEGG functional category definitions (Kanehisa et al., 2016). *Liberibacter* bacteria are largely restricted to the phloem of plants, which is rich in sugars and amino acids, so we hypothesized that the different species of *Liberibacter* would exhibit differences in the number of genes involved in the metabolism of these compounds due to differences in pathogenic ability. For each genome analyzed, we counted the number of genes in each category and investigated the species-specific differences (Figure 5). We found a significant difference in the number of amino acid, energy, and nucleotide metabolism genes between the different species (*p* < 0.001, ANOVA). However, the number of genes involved in carbohydrate metabolism was not significantly different between the species (*p* = 0.13), likely reflecting the importance of carbohydrate metabolism and constraint against losing this function. Isolates from the species *L. crescens* had significantly higher abundance of amino acid and energy biosynthesis related genes compared to all other ‘*Ca. Liberibacter*’ species. In contrast, the Las isolates had a significantly higher number of nucleotide biosynthesis genes compared to Lam, Leu, and Lso isolates.

### 3.5. Genome rearrangement

We also investigated large scale genome rearrangements and changes in genomic synteny across the *Liberibacter* genus, as this provides another way to investigate evolutionary relationships within the genus. Previous work has demontrated extensive genome rearrangement in *Liberibacter* genomes, but only in a handful of genomes (Thapa et al., 2020; Tan et al., 2021). We have used progressiveMauve to preform extended whole genome alignment on two sets of *Liberibacter* genomes: a set of 12 genomes representing the two highest quality genomes from each of the six species and a second set representing the 16 available chromosome-level genome assemblies representing five species of *Liberibacter*: Laf, Lam, Las, Lso, and *L. crescens*.

The alignment of 16 assemblies revealed a total of 136 synteny blocks, or locally collinear blocks (LCBs), across all 16 genomes. An LCB refers to a conserved stretch of the genome with no evidence of rearrangement history. The 136 LCBs spanned a sequence length of 1,167,307 bp and had an average pairwise identity of 60.5% across isolates (Table 3). We compared the phylogenetic relationships in the tree built from the core gene alignment to a phylogeny built from pairwise distances inferred from the Mauve whole genome alignment (Supplementary Figure S4). Considering just the tips of the tree, 12/16 of the isolates maintained the same position in the tree; all four isolates with different positions were Las isolates. The congruence between the two trees indicates that the core gene alignment and whole genome alignment recapitulate similar phylogenetic histories, but with evidence for phylogenetically informative rearrangements in Las.

To investigate the genus wide rearrangement history, we also performed progressiveMauve alignments with the set of 12 most-contiguous genomes representing all six species and within individual species (Table 3). We performed the alignment several times: once all together with all 12 genomes and again once for each species individually to compare the amount of LCBs, which reflects the amount of rearrangement (Darling et al., 2007). The alignment with all 12 of the representative genomes identified156 LCBs spanning a total of 1,148,204 bp, with a pairwise sequence identity of 44.6%. As previously described, we compared the phylogeny built from this whole genome alignment to the phylogeny built from the core gene sequences (Supplementary Figure S5), and we found no differences between the two trees.

Additionally, we performed progressiveMauve alignments for each species individually in order to investigate any species-specific rearrangements and to compare LCB counts within and between species. In contrast to genus-wide analysis, species-specific mauve analysis retrieved very few LCBs – i.e., an average of 11.25 within species. Lso isolates had the most synteny blocks with 46 LCBs identified, followed by the Lam group with 22 LCBs (Table 3). Because the Las group had the most genomes available (*n* = 36) and Mauve is computationally intensive, we performed the progressiveMauve analysis with three different sets of Las genomes: single contig assemblies (*n* = 10), a random set of eight genomes from the entire set of 36 Las genomes, and a random set of eight genomes composed of >1 contig from a set of 26 Las assemblies. The analysis revealed 1, 3, or 11 LCBs depending on the set of genomes analyzed (Table 3). Overall, these analyses indicate that the *Liberibacter* genus has an extensive rearrangement history across species, with 156 synteny blocks shared across the entire genus. As expected, there were fewer LCBs within species, reflecting fewer obvious genome rearrangements within species, but the Lso group was notable for its relative high number of LCBs.

### 3.6. Accessory gene composition and phylogenetic patterns

**Figure 6:**
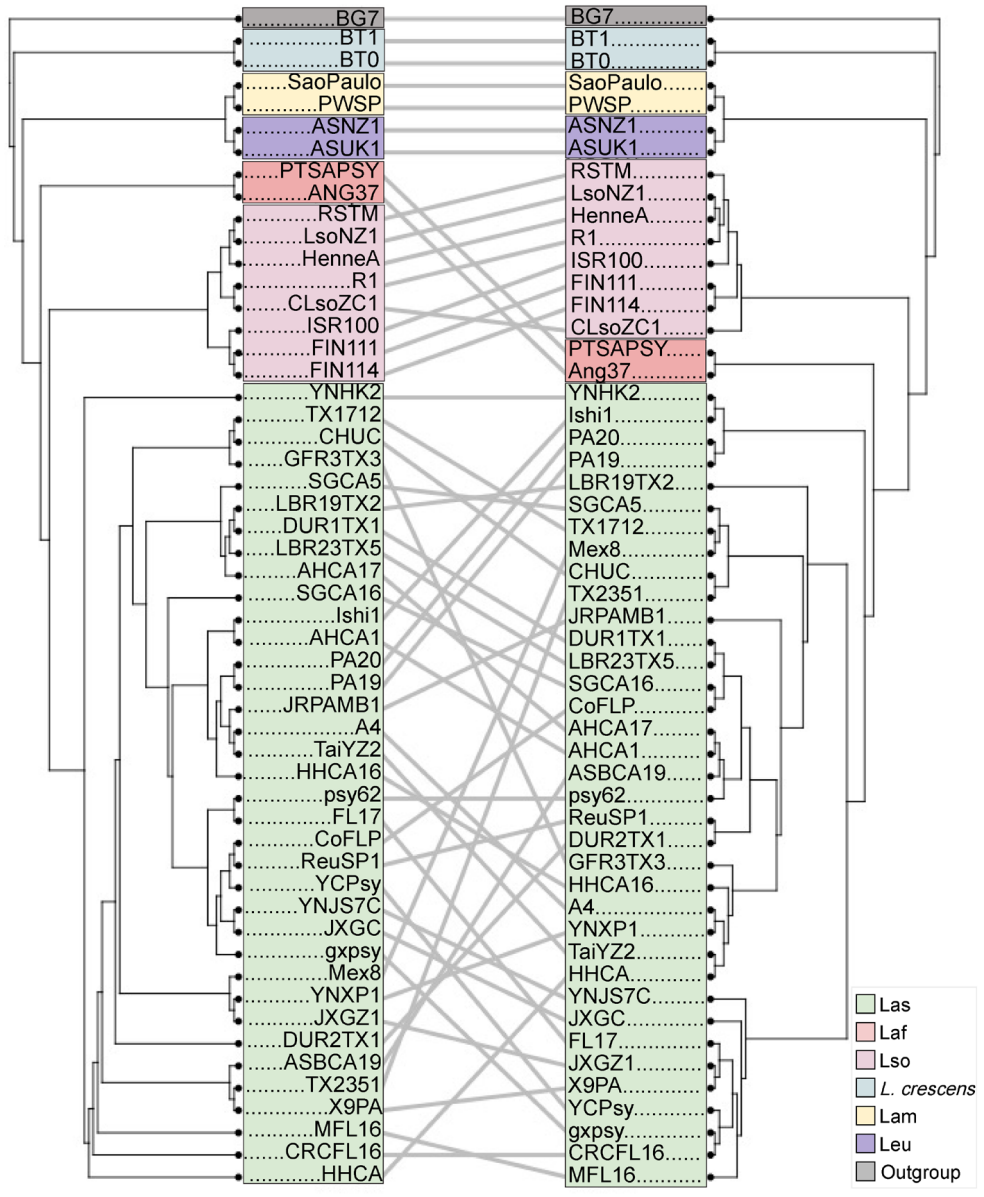
A comparison of a NJ tree based on distances due to accessory gene presence/absence patterns (left) to the maximum likelihood tree based on the core gene alignment (right; Figure 1). As in Figure 1, the isolates are labeled at the tips of the trees, with clades colored according to species. Lines connect the same isolate between the two trees, with angled lines representing topological discordance between phylogenies.

Genome rearrangement, reduction, and recombination is prevalent in the *Liberibacter* genus, and it has been hypothesized to be important towards pathogenicity and virulence evolution (Tan et al., 2021). To better understand these issues, we first investigated the evolutionary patterns of the accessory genes by comparing the core gene phylogeny against a phylogeny based on the accessory gene composition (Figure 6). The accessory gene phylogeny recapitulates a similar clustering pattern as the core gene phylogeny, which has accessions clearly grouped by species. However, the correlation between the two distance matrices used to build each tree was non-significant based on the Mantel test (*p* = 0.086, *R* = 0.158, Mantel Test). This discordance was prominent in the overall species clustering as the placement of the Laf accessions was positioned at a deeper portion of the accessory gene tree compared to the core gene tree. The core gene tree positioned the Laf accessions more closely to Las in a single clade that diverges from the Lso isolates, while the accessory gene tree placed the Laf accessions farther from the Las group and closer to the Leu and Lam clade. The overall pattern of accessory gene content suggests that isolates of the same species have more similar accessory gene content and also that gene exchange between species are not occurring at a rate to alter phylogenetic signal, with the except of the placement of Laf accessions. This statement is supported by ANOSIM, which detected a significant association between accessory gene content and species (ANOSIM *R* = 0.4525, *p* < 2 × 10^−4^).

We next investigated the correlation between the core gene tree and accessory gene tree within Las and Lso, because those groups had >2 accessions to compare. In the Lso clade, the position of 6 of the 8 Lso accessions remained the same across the two trees, suggesting that the core gene nucleotide evolution and accessory gene composition recapitulate similar evolutionary histories (Figure 6). Isolates CLsoZC1 and ISR100 experienced positional changes between the two trees. In the core gene tree, CLsoZC1 diverged from the 7 other Lso accessions; however, in the accessory gene tree, CLsoZC1 formed a group with 4 other Lso isolates. The Lso isolate ISR100 also changed position, as it was originally grouped with LsoNZ1, HenneA, R1, and RSTM in the phylogenetic tree built from the nucleotide core gene alignment, however, in the accessory gene tree, ISR100 formed a group with FIN111 and FIN114. Unlike Lso, the Las group showed extensive changes between the two trees. Most notably, we observed the presence of three clusters within the Las clade based on the core gene alignment, but this clustering pattern was not seen in the tree constructed from accessory gene composition. Altogether, the results suggest that accessory gene content varied by species, leading to similar phylogenetic inference across species based on core genes or gene presence/absence. However, there is enough gene content variation within *Liberibacter* species to create slight differences in the phylogenetic patterns within species.

### 3.7. Measuring selection

To investigate the strength and pattern of selection on individual genes, we estimated the ratio of nonsynonymous mutations to synonymous mutations, referred to as ω or dN/dS. By calculating this ratio, we can identify genes with evidence of positive selection (ω > 1.0) which suggests that these genes may be relevant towards pathogenicity and virulence (Mitchell et al., 2012; Aleru and Barber, 2020). Using the codeml models available through the program PAML, we applied a series of nucleotide substitution models on single-copy core and accessory genes identified in the pan-genome analysis. We tested for positive selection using two methods: either globally across the entire phylogeny (referred to as the global test) or across codon sites (referred to as sites models). In total, we performed these analyses on 1,698 qualifying genes (436 core genes and 1,262 accessory genes; see Methods).

**Figure 7:**
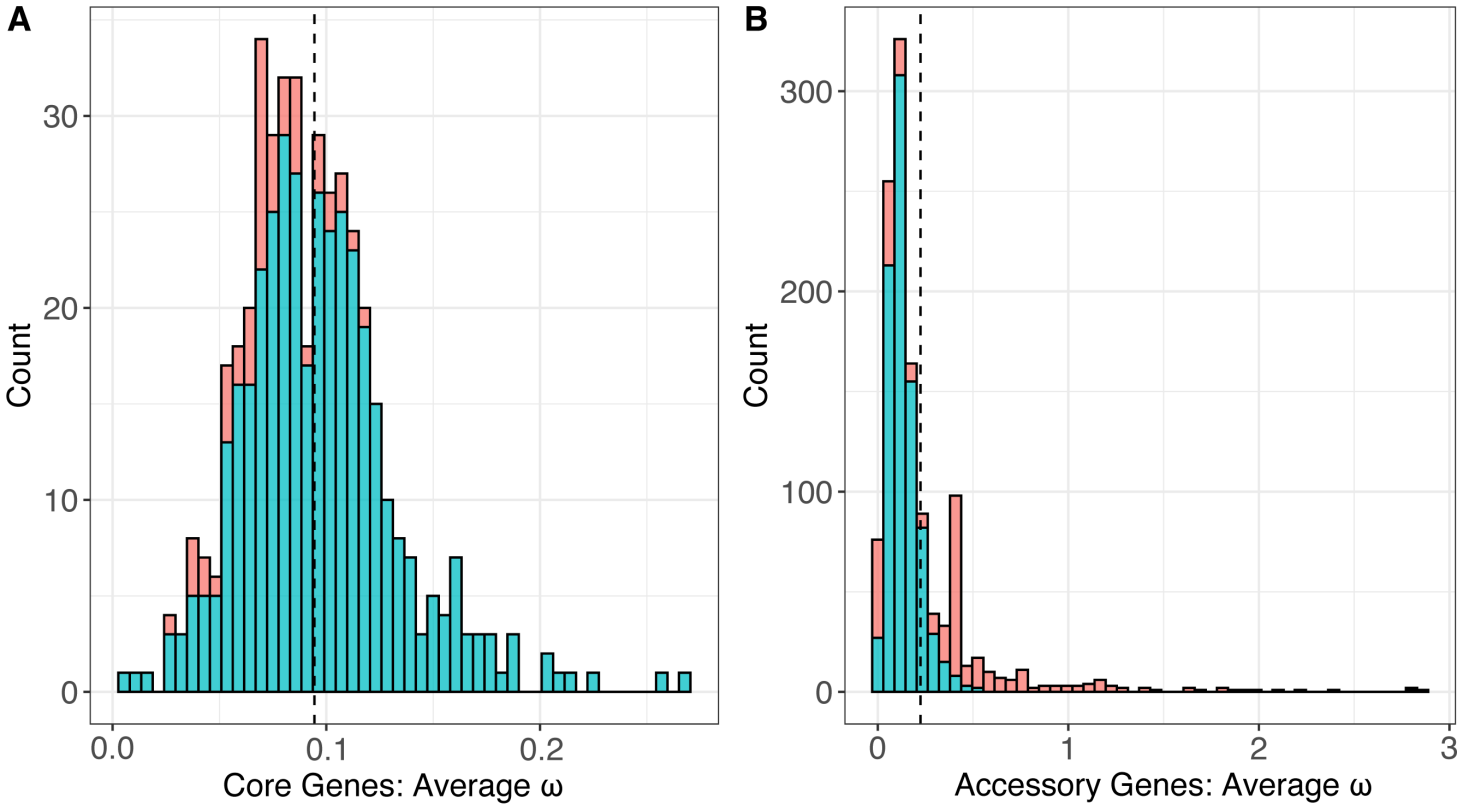
The distribution of estimated values of ω, or dN/dS, under the One-Ratio model (M0) which estimated a single value of ω across an entire gene tree for the set of **(A)** 436 core genes and **(B)** 2,639 accessory genes.

Using the global test, we estimated the single, average ω value for each gene across the phylogeny of the genus. This method assumes that the value of ω is constant across all of the branches in the gene tree and across the individual codons in the nucleotide alignment. For the 436 core genes, estimates of ω ranged from 0.00649 to 0.26883 with an average of 0.09442 (Figure 7A). All of the core genes had estimates of ω less than 1.0, and the vast majority (385/436) had ω estimates significantly less than 1.0 (*p* < 0.01, FDR correction), indicating a pervasive signal of selective constraint on amino acid replacing mutations. For the 1,262 accessory genes, ω ranged from 0.001 to 2.85930 with a mean of 0.22465 (Figure 7B). Among these, we identified 36 accessory genes with estimates of ω > 1, suggesting the possibility of positive selection, but only two had ω estimates significantly higher than 1.0 (*p* < 0.01, FDR correction). We compared the values of average ω estimated for the core and accessory genes; the two groups were significantly different, with accessory genes having significantly higher ω on average (*p* < 2.2 × 10^−16^, *T*-test).

We also applied the sites models in codeml, which test for variation in ω among codons in the gene sequences and also identify whether any sites have a history of positive selection. This represents a less conservative method than the global test to search for positive selection. The sites models are a group of nested models, so we first compared sites model M0 against sites model M3. Sites model M0 represents the null hypothesis that there is a single ω value for all sites in the gene sequence, while sites model M3 permits ω to vary among sites. The genes that were significant for the likelihood ratio test between sites model M0 and M3 were then analyzed under the sites models M2a and M1a which tests for a history of positive selection across codon sites. All but one of the core genes rejected the null hypothesis that a single ω value exists across all sites in the sequence, suggesting variable ω across codons (*p* < 0.01, FDR correction). (The gene *ftcR* was the one non-significant exception.) However, we did not find any core genes that had evidence of a history of positive selection in any of their codons based on M2a-M1a comparisons. The accessory genes displayed contrasting dynamics, because only 64% of the accessory genes (806/1,262) rejected the null that a single ω value exists across all sites in the M0–M3 comparison. We investigated the 806 genes further using the M2a-M1a model and found that 52 accessory genes had significant evidence of a history of positive selection among sites in the sequence (*p* < 0.01, FDR correction). Among these 52 accessory genes, ten also had evidence of positive selection via the global test, as indicated by estimates of ω > 1 (Table 4).

**Table 4:** Genes with evidence of positive selection based on both the global and sites models.

Gene label	BlastP	CD blast	eggNOG-mapper description	COG	COG description
group_2457	von Willebrand factor type A, *tadE/tadG*	vWFA	Oxidoreductase activity	IU	Lipid transport and metabolism and Intracellular trafficking, secretion, and vesicular transport
group_2824	von Willebrand factor type A, *tadE/tadG*	*tadE*	von Willebrand factor type A domain	S	Function unknown
group_2562	von Willebrand factor type A	*tadG*	PFAM TadE family protein	U	Intracellular trafficking, secretion, and vesicular transport
group_225	Hypothetical protein	NA	Nuclear chromosome segregation	D	Cell cycle control, cell division, chromosome partitioning
group_2579	Metallophosphoesterase	NA	NA	NA	NA
group_2831	HPE1 family effector	NA	NA	NA	NA
group_1354	Hypothetical protein	NA	NA	NA	NA
group_1502	Hypothetical protein	NA	NA	NA	NA
group_2564	Hypothetical protein	NA	NA	NA	NA
group_2569	Hypothetical protein	NA	NA	NA	NA

## 4. Discussion

We have investigated genetic diversity and genome evolution within the *Liberibacter* genus. Our work is based on 52 whole genome sequences from six different species, representing a much-expanded sample relative to previous work (Thapa et al., 2020; Tan et al., 2021). Our set of genomes included five different non-culturable species of ‘*Ca.* Liberibacter*’* and the culturable *L. crescens*. Like previous work, our analyses show that *Liberibacter* species are highly divergent based on nucleotide identity, gene content, and rearrangement history. However, our work also yields novel insights into the evolutionary history of genomes, including the prevalence of genome rearrangement and gene/presence absence within vs. between species and also into the prevalence of positive selection, particularly in accessory genes.

To help understand species’ designations, we measured average nucleotide identity (ANI) within and between species. The average inter-species ANI was 76.2%. Previous work has suggested that prokaryotic species boundaries have ANI values <83% (Jain et al., 2018), so ANI values within *Liberibacter* illustrate verify that they are, indeed, distinct species by this measure. There were also significant differences in intra-species ANI, with Lso isolates having the lowest ANI at 98.17%. Despite the lower ANI calculated for Lso isolates, the ANI value is still within the accepted range for prokaryotic species demarcation, which is generally accepted as >95% (Jain et al., 2018), although such a universal genetic boundary for prokaryotes is debated (Murray G. G. R. et al., 2021). Lso has the largest known host range compared to the other pathogenic species in the genus and, interestingly, Lso was the only species in the analysis to have an ANI below 99%, in addition to having the lowest percent identity (89%) across its LCBs in the Mauve analysis (Table 3). While there are limitations to the dataset due to uneven sampling, these results are unlikely not due to higher sampling alone. Although the Las group has the most genomes available (*n* = 36), it has a much narrower range of pairwise ANI values compared to Lso isolates (*n* = 8; Supplementary Figure S2). We hypothesize that the high genetic diversity within Lso partially reflects biological differences, particularly the wide host range of the Lso group. Lso infects solanaceous plants, but unlike Las which predominantly infects citrus (family *Rutaceae*), Lso has also recently been isolated from vectors known to associate with other plants in the families *Oleaceae* and *Polygonaceae* (Haapalainen et al., 2020; Wamonje et al., 2022). Previous studies using different Lso samples have identified divergent haplotypes between Lso isolates, corroborating our inference that Lso harbors high genomic diversity (Wang J. et al., 2017).

Our analyses of core gene content also highlight divergence within and between the different *Liberibacter* species. Of 8,926 genomes in the pan-genome, we have identified a set of 436 single copy core genes across the genus. The number of core genes retrieved was similar to other studies that have identified core genes or orthologous groups in different sets of *Liberibacter* sequences (Thapa et al., 2020; Hansen et al., 2022), albeit on much smaller genomic samples. On average, our sample of *Liberibacter* genomes had 1,213 predicted coding sequences, and thus the core genes represent 36.0% of the genes found in each genome. This is a relatively small percentage compared to other plant-associated bacteria. For example, genus-wide pan-genome analyses of pathogens like *Xanthomonas* or *Xylella* typically reveal a core genome size that consists of roughly half the genes in the genome (Ariute et al., 2022; Batarseh et al., 2022). Due to the low percentage of core genes (and correspondingly high number of accessory genes) in the pan-genome, we also quantified and compared the species-specific core genes. Not surprisingly, the number of core genes per species was higher than the genus-wide estimate, because we identified an average of 985 core genes per species compared to 436 core genes genus-wide.

We compared phylogenetic relationships between species based on core gene sequences to a tree built from the presence / absence patterns of accessory genes. Ours is the first study, to our knowledge, that has investigated the phylogenetic relationships of *Liberibacter* using accessory gene content, as most studies utilize core genes or a set of conserved marker genes to build phylogenies (Leonard et al., 2012; Wang J. et al., 2017; Haapalainen et al., 2020; Thapa et al., 2020). We have made three sets of observations based on accessory gene analyses. First, several changes occurred between the core and accessory phylogenies, with the most notable being the position of the Laf isolates. Lso is more closely related to Las in the accessory gene phylogeny, but Las and Laf are sister species in the core gene phylogeny (Figure 6). The positional change of Lso reflects an exceptionally high number of shared accessory genes (293) between these two species (Supplementary Figure S3B), compared to 42 shared with Laf and Las. The relatively high number could reflect sampling phenomena, but nonetheless illustrates that many accessory genes can be shared between species. Second, the data hint at intriguing differences in accessory gene content. Compared to the other species with only two genome isolates (Lam and Leu), Laf has a much higher number of accessory genes (Supplementary Figure S3B). Intrigued, we annotated shared genes with eggNog, hoping to gain functional insights into the cause of this observation. We found the majority of unique genes identified in Laf genomes were involved in replication, recombination, and repair, but these functions unfortunately tell us little about potential unique environmental or ecological characteristics that could be driving accessory genome content in Laf. Additional sequencing of complete genomes of the Laf, Lam, and Leu species will be beneficial for better understanding the pattern of accessory genes across the genus. Finally, the accessory gene tree also highlighted differences within Lso isolates by splitting them into two groups that were not seen in the core gene tree. For example, isolates FIN111, FIN114, and ISR100 form their own group based on accessory gene content. Intriguingly, FIN111 and FIN114 were extracted from carrot psyllids and were found to harbor extensive differences in their genome organization (Wang J. et al., 2017). The accessory gene content of those two isolates was similar to ISR100, which was from a different study, a different geographic location and even clustered separately from FIN111 and FIN114 in the core gene tree (Katsir et al., 2018). By investigating a larger amount of *Liberibacter* genomes, our study suggests that the accessory gene content in these two isolates are highly similar relative to their divergence measured by core gene sequences. We again do not know if there are ecological or functional drivers of these shifts in accessory gene content but look forward to future work that addresses these issues.

We also investigated phylogenetic relationships of *Liberibacter* species based on inferred genome rearrangements. We note that the *Liberibacter* genome assemblies are typically fragmented due to the inability to culture and directly sequence the bacteria, therefore extensive analysis of genome rearrangement is limited. Due to this reason, we used a reference genome to align the contigs before performing the Mauve alignment as suggested by the program authors. Similar to previous studies comparing *Liberibacter* genomes, we identified an extensive number of rearrangements (Thapa et al., 2020; Tan et al., 2021). We have found that *Liberibacter* has 156 LCBs genus wide, with a percent identity of 44.6%. However, accounting for the rearrangement history did not change inferred relationships among species (Supplementary Figures S4, S5).

Finally, to investigate global patterns of selection and identify genes experiencing selection and that may be important drivers of pathogenicity, we measured selection in the coding regions of the genome. To do so, we measured dN/dS, or ω, and performed statistical tests of positive selection on a set of 1,698 genes (436 core genes and 1,262 accessory genes) using the codeml models through PAML. A recent study investigating *Liberibacter* genomes and the newly identified species *Ca.* L. capsica, measured dN/dS in a set of 433 orthologous genes in the genus and identified 21 proteins with high dN values that may be involved in pathogenesis and host–microbe interactions, but this study did not perform any statistical tests for positive selection (Hansen et al., 2022). Another study applied tests for positive selection on a genus-wide sample of *Liberibacter* core genes, based on a smaller sample of isolates, but did not estimate dN/dS in accessory genes (Chen et al., 2020). Here, we calculated a single value of ω (dN/dS) for each gene which represented a conservative test of selection globally across all genes and all sites in the gene sequence. The global test revealed the 436 *Liberibacter* core genes were under purifying selection across the genus, because all genic estimates of ω were < 1.0 and most estimates were significantly <1.0 (Figure 7). The trend of pervasive purifying selection is not especially surprising, because it holds for core genes appears across multiple bacterial species with different life history traits, including the xylem-limited plant pathogen *Xylella fastidiosa* and the human pathogen *Helicobacter pylori* (Rubio and Pérez-Pulido, 2021; Batarseh et al., 2022).

The set of 1,698 accessory genes displayed significantly different estimates of ω compared to the core gene set, because ω was higher on average compared to core genes (average ω for accessory genes = 0.225, average ω for core genes = 0.094). We note, however, that the estimates of ω for accessory genes have reduced statistical power relative to the core genes due to the smaller sample sizes for the gene alignments (*n* = 4 to 50 for accessory genes compared to *n* = 51 to 53 for core genes). Notably, lower values of dN/dS across accessory genes in *Liberibacter* followed patterns similar patterns to other pathogenic bacteria (Rubio and Pérez-Pulido, 2021; Batarseh et al., 2022).

We also documented genes with significant statistical evidence of positive selection (dN/dS > 1.0), because this genic set bears the potential signal of arms-race dynamics and may thus have a role in pathogenicity (Anderson et al., 2010). Some signatures of positive selection may be hidden with the global test, because the global test is performed by averaging dN/dS across all sites and branches in the phylogeny. To complement the more conservative global test, we also calculated dN/dS using sites models, a group of nested models that permit dN/dS to vary across sites in a sequence. Similar to Chen et al. (2020), our analysis did suggest that the core genes in *Liberibacter* have evidence of variable dN/dS among sites, although for core genes we found no evidence that variability in dN/dS signaled positive selection. In contrast, this model identified a set of 52 accessory genes that had significant evidence of a history of positive selection among sites. Together, the global test and the sites models identified a set of 78 accessory genes with evidence of positive selection but no core genes with sufficient evidence. Of these 78 genes, ten also had dN/dS values >1.0 based on their complete phylogenies. Our work also documents that *Liberibacter* has a similar proportion of core and accessory genes with a history of positive selection compared to *X. fastidiosa,* which is also an insect vectored plant pathogen (Batarseh et al., 2022). It also drives home the point that most genes inferred to be under positive-selection – and thus subject to arms-race dynamics by this specific criterion – are found in the accessory gene complement.

We also caution that positive selection analyses are subject to false positive and are dependent on particular features such as sample set, criteria for determining homology, and sequence alignments. Nonetheless, given that ten genes were implicated to be under positive selection under both models, we explored their potential functions in more detail (Table 4). All ten genes were originally annotated as hypothetical proteins by Prokka, so we utilized the NCBI conserved domain Blast and the eggNOG mapper to glean additional annotation information. We ultimately identified putative annotations and functional information for six of the ten genes. Three of these genes were accessory genes found only in Lso isolates (considered a core gene within Lso) and were annotated as proteins related to the Tad complex and having Von Willebrand Factor Type A protein domains. The Tad pilus complex has been implicated in host cell interactions and the uptake of DNA (Andrade and Wang, 2019) and the Von Willebrand Factor Type A protein domain is important for cell adhesion, migration, and signal transduction (Steinert et al., 2020). Interestingly, genes in the Tad complex have been previously implicated as being highly divergent in the ‘*Ca.* L. capsica’ genome based on high values of dN (Hansen et al., 2022). Moreover, through Blast analyses we recovered annotation evidence for two other genes: group_2579 was annotated as a metallophosphoesterase and group_2831 as hypothetical protein effector 1 (HPE1). HPE1 has been experimentally shown to suppress plant effector-triggered immunity in Lso infection (Kan et al., 2021). Our results corroborate the potential importance of this effector family based on a history of positive selection. All ten genes are excellent candidates for further functional analysis, such as measuring immune response in plant cells (Chen et al., 2020).

**Figure 8:**
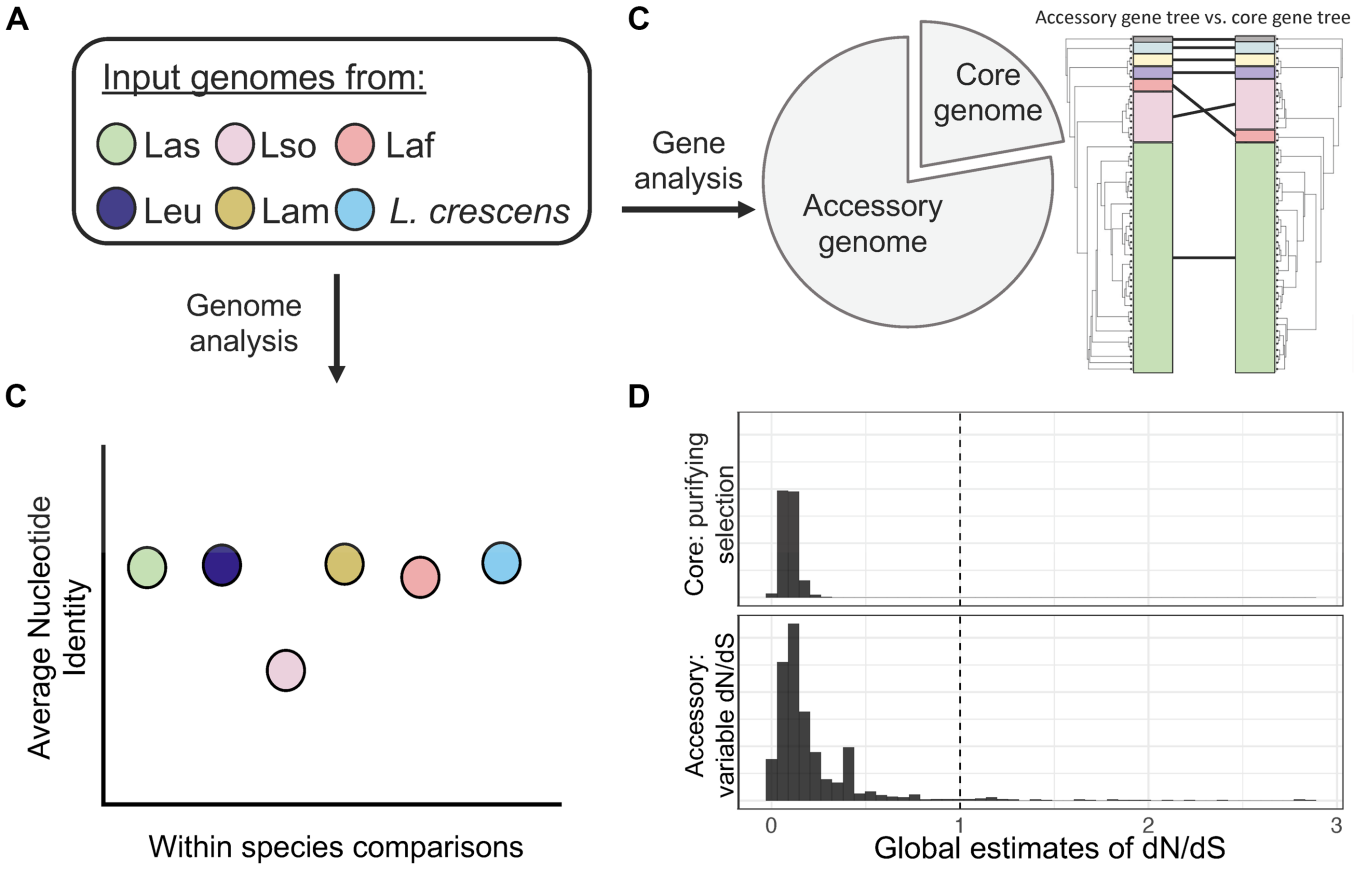
A summary figure of the overarching comparative genomics outcomes from this study. **(A)** Our pipeline consisted of investigating whole genomes of *Liberibacter* from six different species. **(B)** Using whole genomes, we found that Lso had the lowest ANI within species, highlighting its high genetic diversity. **(C)** Pan-genome analysis identified a set of 436 core genes and 1,698 accessory genes. We investigated the phylogenetic relationships by building a phylogenetic tree using the core gene nucleotide alignment and comparing it to a tree built from the presence absence patterns of accessory genes. The comparison revealed different topologies between the two trees as the Laf species clustered more closely with Las based on core gene sequences but was farther from Las based on accessory gene content. **(D)** We estimated dN/dS across core and accessory genes to investigate for signatures of selection. We found a pattern of purifying selection across core genes, but variable dynamics across the set of accessory genes with several inferred to have experienced positive selection.

Overall, our analysis of whole genomes from six different *Liberibacter* species has genome diversity in terms of sequence identity, accessory gene content, genome rearrangement and an apparent history of positive selection (Figure 8). We cannot ignore, however, that our results have limitations due to sampling issues that need to be addressed in future research. The majority of our isolates in the analysis were Las; it will be important to sequence more genomes in the other species, especially *L. crescens*, Laf, Lam, and Leu, to obtain better representation across the genus. Additionally, more species of *Liberibacter* are being proposed based on new information (Kwak et al., 2021). Additional full genome sequencing of the newly proposed species, such as ‘*Ca.* L. capsica’, should also be performed and investigated to include in a comparative genomics framework. To fully recapitulate genomic rearrangements, it is crucial to have future work based on closed genome assemblies or long-read sequencing data, but these are inherently challenging tasks due to the inability to culture pathogenic *Liberibacter*.

## Data availability statement

The original contributions presented in the study are included in the article/Supplementary material, further inquiries can be directed to the corresponding author.

## Author contributions

AM-C and BG contributed to conception and design of the study. SB downloaded and organized the genome files from public databases. TB and SB performed bioinformatic and statistical analyses. TB prepared the first draft of the manuscript and figures. All authors contributed to the article and approved the submitted version.

## Funding

TB was supported by the National Science Foundation Postdoctoral Research Fellowship in Biology (#2209111). BG was supported by the National Science Foundation grant #1741627.

## Conflict of interest

The authors declare that the research was conducted in the absence of any commercial or financial relationships that could be construed as a potential conflict of interest.

## Publisher’s note

All claims expressed in this article are solely those of the authors and do not necessarily represent those of their affiliated organizations, or those of the publisher, the editors and the reviewers. Any product that may be evaluated in this article, or claim that may be made by its manufacturer, is not guaranteed or endorsed by the publisher.

## Footnotes

^1^ https://github.com/gpertea/cdbfasta

^2^ http://abacus.gene.ucl.ac.uk/software/pamlFAQs.pdf

## References

[ref1] Aguileta G. Refrégier G. Yockteng R. Fournier E. Giraud T. (2009). Rapidly evolving genes in pathogens: methods for detecting positive selection and examples among fungi, bacteria, viruses and protists. Infect. Genet. Evol. 9, 656–670. doi: 10.1016/j.meegid.2009.03.010, PMID: 19442589

[ref2] Albrecht U. Bowman K. D. (2009). Candidatus *Liberibacter* asiaticus and Huanglongbing effects on Citrus seeds and seedlings. HortScience 44, 1967–1973. doi: 10.21273/HORTSCI.44.7.1967

[ref3] Aleru O. Barber M. F. (2020). Battlefronts of evolutionary conflict between bacteria and animal hosts. PLoS Pathog. 16:e1008797. doi: 10.1371/journal.ppat.1008797, PMID: 32941529 PMC7498106

[ref4] Alquézar B. Carmona L. Bennici S. Peña L. (2021). Engineering of citrus to obtain huanglongbing resistance. Curr. Opin. Biotechnol. 70, 196–203. doi: 10.1016/j.copbio.2021.06.003, PMID: 34198205

[ref5] Anderson J. P. Gleason C. A. Foley R. C. Thrall P. H. Burdon J. B. Singh K. B.. (2010). Plants versus pathogens: an evolutionary arms race. Functional Plant Biol. 37, 499–512. doi: 10.1071/FP09304, PMID: 21743794 PMC3131095

[ref6] Andrade M. Wang N. (2019). The tad pilus apparatus of ‘Candidatus *Liberibacter* asiaticus’ and its regulation by VisNR. MPMI 32, 1175–1187. doi: 10.1094/MPMI-02-19-0052-R, PMID: 30925227

[ref7] Ariute J. C. Rodrigues D. L. N. de Castro Soares S. Azevedo V. Benko-Iseppon A. M. Aburjaile F. F. (2022). Comparative genomic analysis of Phytopathogenic Xanthomonas species suggests high level of genome plasticity related to virulence and host adaptation. Bacteria 1, 218–241. doi: 10.3390/bacteria1040017

[ref8] Bassanezi R. B. Montesino L. H. Gasparoto M. C. G. Bergamin Filho A. Amorim L. (2011). Yield loss caused by huanglongbing in different sweet orange cultivars in São Paulo, Brazil. Eur. J. Plant Pathol. 130, 577–586. doi: 10.1007/s10658-011-9779-1

[ref9] Batarseh T. N. Morales-Cruz A. Ingel B. Roper M. C. Gaut B. S. (2022). Using genomes and evolutionary analyses to screen for host-specificity and positive selection in the plant pathogen *Xylella fastidiosa*. Appl. Environ. Microbiol. 88:e0122022. doi: 10.1128/aem.01220-22, PMID: 36094203 PMC9499020

[ref10] Boc A. Diallo A. B. Makarenkov V. (2012). T-REX: a web server for inferring, validating and visualizing phylogenetic trees and networks. Nucleic Acids Res. 40, W573–W579. doi: 10.1093/nar/gks485, PMID: 22675075 PMC3394261

[ref11] Bosi E. Fani R. Fondi M. (2015). “Defining Orthologs and Pangenome size metrics” in Bacterial Pangenomics: Methods and Protocols Methods in Molecular Biology. eds. Mengoni A. Galardini M. Fondi M. (New York, NY: Springer), 191–202. 10.1007/978-1-4939-1720-4_13 25343867

[ref12] Bové J. M. (2006). Huanglongbing: a destructive, newly-emerging, century-old disease of Citrus. J. Plant Pathol. 88, 7–37.

[ref13] Castresana J. (2000). Selection of conserved blocks from multiple alignments for their use in phylogenetic analysis. Mol. Biol. Evol. 17, 540–552. doi: 10.1093/oxfordjournals.molbev.a026334, PMID: 10742046

[ref14] Chase A. B. Gomez-Lunar Z. Lopez A. E. Li J. Allison S. D. Martiny A. C.. (2018). Emergence of soil bacterial ecotypes along a climate gradient. Environ. Microbiol. 20, 4112–4126. doi: 10.1111/1462-2920.14405, PMID: 30209883

[ref15] Chen Y. Bendix C. Lewis J. D. (2020). Comparative genomics screen identifies microbe-associated molecular patterns from ‘Candidatus *Liberibacter’* spp. that elicit immune responses in plants. MPMI 33, 539–552. doi: 10.1094/MPMI-11-19-0309-R, PMID: 31790346

[ref16] Coletta-Filho H. D. Carlos E. F. Lotto L. L. Luciane F. C. Alves K. C. S. Pereira M. A. R.. (2010). Prevalence of Candidatus *Liberibacter* spp. in HLB-Diseased Citrus Plants in São Paulo State, Brazil. International Organization of Citrus Virologists Conference Proceedings (1957–2010) 17.

[ref17] Darling A. C. E. Mau B. Blattner F. R. Perna N. T. (2004). Mauve: multiple alignment of conserved genomic sequence with rearrangements. Genome Res. 14, 1394–1403. doi: 10.1101/gr.2289704, PMID: 15231754 PMC442156

[ref18] Darling A. E. Treangen T. J. Messeguer X. Perna N. T. (2007). “Analyzing patterns of microbial evolution using the mauve genome alignment system” in *Comparative genomics* Methods in Molecular Biology™. ed. Bergman N. H. (Totowa, NJ: Humana Press), 135–152. 10.1007/978-1-59745-515-2_10 18025691

[ref19] Dominguez-Mirazo M. Jin R. Weitz J. S. (2019). Functional and comparative genomic analysis of integrated prophage-like sequences in “Candidatus *Liberibacter* asiaticus.”. mSphere 4:e00409-19. doi: 10.1128/mSphere.00409-19 31722990 PMC6854039

[ref21] Gao F. Wu B. Zou C. Bao Y. Li D. Yao W.. (2022). Genetic diversity of “Candidatus *Liberibacter* asiaticus” based on four hypervariable genomic regions in China. Microbiology Spectrum 10:e02622-22. doi: 10.1128/spectrum.02622-22, PMID: 36409071 PMC9769890

[ref22] Haapalainen M. Latvala S. Wickström A. Wang J. Pirhonen M. Nissinen A. I. (2020). A novel haplotype of ‘Candidatus *Liberibacter* solanacearum’ found in Apiaceae and Polygonaceae family plants. Eur. J. Plant Pathol. 156, 413–423. doi: 10.1007/s10658-019-01890-0

[ref23] Hansen A. K. Sanchez A. N. Kwak Y. (2022). Divergent host-microbe interaction and pathogenesis proteins detected in recently identified *Liberibacter* species. Microbiol. Spectrum 10:e02091-22. doi: 10.1128/spectrum.02091-22, PMID: 35900091 PMC9430466

[ref100] Hajri A. Loiseau M. Cousseau-Suhard P. M. Renaudin I. Gentit P. (2017). Genetic Characterization of ‘Candidatus Liberibacter solanacearum’ Haplotypes Associated with Apiaceous Crops in France. Plant Disease. 101:1383–1390. doi: 10.1094/PDIS-11-16-1686-RE, PMID: 30678593

[ref24] Huerta-Cepas J. Forslund K. Coelho L. P. Szklarczyk D. Jensen L. J. von Mering C.. (2017). Fast genome-wide functional annotation through Orthology assignment by eggNOG-mapper. Mol. Biol. Evol. 34, 2115–2122. doi: 10.1093/molbev/msx148, PMID: 28460117 PMC5850834

[ref25] Huerta-Cepas J. Szklarczyk D. Heller D. Hernández-Plaza A. Forslund S. K. Cook H.. (2019). eggNOG 5.0: a hierarchical, functionally and phylogenetically annotated orthology resource based on 5090 organisms and 2502 viruses. Nucleic Acids Res. 47, D309–D314. doi: 10.1093/nar/gky1085, PMID: 30418610 PMC6324079

[ref26] Jagoueix S. Bove J.-M. Garnier M. (1994). The phloem-limited bacterium of greening disease of Citrus is a member of the α subdivision of the Proteobacteria. Int. J. Syst. Evol. Microbiol. 44, 379–386. doi: 10.1099/00207713-44-3-379, PMID: 7520729

[ref27] Jain M. Cai L. Fleites L. A. Munoz-Bodnar A. Davis M. J. Gabriel D. W. (2019). *Liberibacter* crescens is a cultured surrogate for functional genomics of uncultured pathogenic ‘Candidatus *Liberibacter*’ spp. and is naturally competent for transformation. Phytopathology 109, 1811–1819. doi: 10.1094/PHYTO-04-19-0129-R, PMID: 31090497

[ref28] Jain C. Rodriguez-R L. M. Phillippy A. M. Konstantinidis K. T. Aluru S. (2018). High throughput ANI analysis of 90K prokaryotic genomes reveals clear species boundaries. Nat. Commun. 9:5114. doi: 10.1038/s41467-018-07641-9, PMID: 30504855 PMC6269478

[ref29] Kan C.-C. Mendoza-Herrera A. Levy J. Hull J. J. Fabrick J. A. Tamborindeguy C. (2021). HPE1, an effector from Zebra Chip pathogen interacts with tomato proteins and perturbs Ubiquitinated protein accumulation. Int. J. Mol. Sci. 22:9003. doi: 10.3390/ijms22169003, PMID: 34445707 PMC8396652

[ref30] Kanehisa M. Sato Y. Morishima K. (2016). BlastKOALA and GhostKOALA: KEGG tools for functional characterization of genome and metagenome sequences. J. Mol. Biol. 428, 726–731. doi: 10.1016/j.jmb.2015.11.006, PMID: 26585406

[ref31] Katsir L. Zhepu R. Santos Garcia D. Piasezky A. Jiang J. Sela N.. (2018). Genome analysis of haplotype D of Candidatus *Liberibacter* Solanacearum. Front. Microbiol. 9:2933. doi: 10.3389/fmicb.2018.02933, PMID: 30619106 PMC6295461

[ref32] Kwak Y. Sun P. Meduri V. R. Percy D. M. Mauck K. E. Hansen A. K. (2021). Uncovering symbionts across the psyllid tree of life and the discovery of a new *Liberibacter* species, “Candidatus” *Liberibacter* capsica. Front. Microbiol. 12:739763. doi: 10.3389/fmicb.2021.739763, PMID: 34659173 PMC8511784

[ref33] Lee I. Ouk Kim Y. Park S.-C. Chun J. (2016). OrthoANI: an improved algorithm and software for calculating average nucleotide identity. Int. J. Syst. Evol. Microbiol. 66, 1100–1103. doi: 10.1099/ijsem.0.000760, PMID: 26585518

[ref34] Leonard M. T. Fagen J. R. Davis-Richardson A. G. Davis M. J. Triplett E. W. (2012). Complete genome sequence of *Liberibacter* crescens BT-1. Stand. Genomic Sci. 7, 271–283. doi: 10.4056/sigs.3326772, PMID: 23408754 PMC3569387

[ref35] Letunic I. Bork P. (2021). Interactive tree of life (iTOL) v5: an online tool for phylogenetic tree display and annotation. Nucleic Acids Res. 49, W293–W296. doi: 10.1093/nar/gkab301, PMID: 33885785 PMC8265157

[ref36] Levy J. G. Gross R. Mendoza-Herrera A. Tang X. Babilonia K. Shan L.. (2020). Lso-HPE1, an effector of ‘Candidatus *Liberibacter* solanacearum’, can repress plant immune response. Phytopathology® 110, 648–655. doi: 10.1094/PHYTO-07-19-0252-R, PMID: 31697198

[ref37] Liefting L. W. Sutherland P. W. Ward L. I. Paice K. L. Weir B. S. Clover G. R. G. (2009). A new ‘Candidatus *Liberibacter*’ species associated with diseases of Solanaceous crops. Plant Dis. 93, 208–214. doi: 10.1094/PDIS-93-3-0208, PMID: 30764179

[ref38] Ma W. Pang Z. Huang X. Xu J. Pandey S. S. Li J.. (2022). Citrus Huanglongbing is a pathogen-triggered immune disease that can be mitigated with antioxidants and gibberellin. Nat. Commun. 13:529. doi: 10.1038/s41467-022-28189-9, PMID: 35082290 PMC8791970

[ref39] Madeira F. Park Y. M. Lee J. Buso N. Gur T. Madhusoodanan N.. (2019). The EMBL-EBI search and sequence analysis tools APIs in 2019. Nucleic Acids Res. 47, W636–W641. doi: 10.1093/nar/gkz268, PMID: 30976793 PMC6602479

[ref40] McDonald B. A. Stukenbrock E. H. (2016). Rapid emergence of pathogens in agro-ecosystems: global threats to agricultural sustainability and food security. Philos. Trans. R. Soc. Lond. Ser. B Biol. Sci. 371:20160026. doi: 10.1098/rstb.2016.0026, PMID: 28080995 PMC5095548

[ref41] Merfa M. V. Naranjo E. Shantharaj D. De La Fuente L. (2022). Growth of ‘Candidatus *Liberibacter* asiaticus’ in commercial grapefruit juice-based media formulations reveals common cell density-dependent transient behaviors. Phytopathology 112, 131–144. doi: 10.1094/PHYTO-06-21-0228-FI, PMID: 34340531

[ref42] Merfa M. V. Pérez-López E. Naranjo E. Jain M. Gabriel D. W. De La Fuente L. (2019). Progress and obstacles in culturing ‘Candidatus *Liberibacter* asiaticus’, the bacterium associated with Huanglongbing. Phytopathology 109, 1092–1101. doi: 10.1094/PHYTO-02-19-0051-RVW, PMID: 30998129

[ref43] Mitchell P. S. Patzina C. Emerman M. Haller O. Malik H. S. Kochs G. (2012). Evolution-guided identification of antiviral specificity determinants in the broadly acting interferon-induced innate immunity factor MxA. Cell Host Microbe 12, 598–604. doi: 10.1016/j.chom.2012.09.005, PMID: 23084925 PMC3540999

[ref44] Mora V. Ramasamy M. Damaj M. B. Irigoyen S. Ancona V. Ibanez F.. (2021). Potato Zebra Chip: an overview of the disease, control strategies, and prospects. Front. Microbiol. 12:700663. doi: 10.3389/fmicb.2021.700663, PMID: 34367101 PMC8339554

[ref45] Morris J. Shiller J. Mann R. Smith G. Yen A. Rodoni B. (2017). Novel ‘Candidatus *Liberibacter*’ species identified in the Australian eggplant psyllid, Acizzia solanicola. Microb. Biotechnol. 10, 833–844. doi: 10.1111/1751-7915.12707, PMID: 28387006 PMC5481521

[ref46] Moulana A. Anderson R. E. Fortunato C. S. Huber J. A. (2020). Selection is a significant driver of gene gain and loss in the Pangenome of the bacterial genus Sulfurovum in geographically distinct Deep-Sea hydrothermal vents. mSystems 5:5. doi: 10.1128/mSystems.00673-19, PMID: 32291353 PMC7159903

[ref101] Munyaneza J. E. Sengoda V. G. Crosslin J. M. Garzón-Tiznado J. A. Cardenas-Valenzuela O. G. (2009). First Report of “Candidatus Liberibacter solanacearum” in Tomato Plants in México. Plant Disease. 93:1076. doi: 10.1094/PDIS-93-10-1076A, PMID: 30754366

[ref47] Munyaneza J. E. Fisher T. W. Sengoda V. G. Garczynski S. F. Nissinen A. Lemmetty A. (2010). First report of “Candidatus *Liberibacter* solanacearum” associated with psyllid-affected carrots in Europe. Plant Dis. 94:639. doi: 10.1094/PDIS-94-5-0639A, PMID: 30754456

[ref48] Murray G. G. R. Charlesworth J. Miller E. L. Casey M. J. Lloyd C. T. Gottschalk M.. (2021). Genome reduction is associated with bacterial pathogenicity across different scales of temporal and ecological divergence. Mol. Biol. Evol. 38, 1570–1579. doi: 10.1093/molbev/msaa323, PMID: 33313861 PMC8042751

[ref49] Murray C. S. Gao Y. Wu M. (2021). Re-evaluating the evidence for a universal genetic boundary among microbial species. Nat. Commun. 12:4059. doi: 10.1038/s41467-021-24128-2, PMID: 34234129 PMC8263626

[ref50] Oksanen J. Guillaume Blanchet F. Friendly M. Kindt R. Legendre P. McGlinn D.. (2020). Vegan: Community Ecology Package. Available at: https://CRAN.R-project.org/package=vegan.

[ref51] Paradis E. Claude J. Strimmer K. (2004). APE: analyses of Phylogenetics and evolution in R language. Bioinformatics 20, 289–290. doi: 10.1093/bioinformatics/btg412, PMID: 14734327

[ref52] Parks D. H. Imelfort M. Skennerton C. T. Hugenholtz P. Tyson G. W. (2015). CheckM: assessing the quality of microbial genomes recovered from isolates, single cells, and metagenomes. Genome Res. 25, 1043–1055. doi: 10.1101/gr.186072.114, PMID: 25977477 PMC4484387

[ref53] R Core Team (2021). R: A Language and Environment for Statistical Computing. R Foundation for Statistical Computing, Vienna, Austria.

[ref54] Raddadi N. Gonella E. Camerota C. Pizzinat A. Tedeschi R. Crotti E.. (2011). ‘Candidatus *Liberibacter* europaeus’ sp. nov. that is associated with and transmitted by the psyllid Cacopsylla pyri apparently behaves as an endophyte rather than a pathogen. Environ. Microbiol. 13, 414–426. doi: 10.1111/j.1462-2920.2010.02347.x, PMID: 21040355

[ref55] Reynaud B. Turpin P. Molinari F. M. Grondin M. Roque S. Chiroleu F.. (2022). The African citrus psyllid Trioza erytreae: an efficient vector of Candidatus *Liberibacter* asiaticus. Front. Plant Sci. 13:1089762. doi: 10.3389/fpls.2022.1089762, PMID: 36618633 PMC9815554

[ref56] Ristaino J. B. Anderson P. K. Bebber D. P. Brauman K. A. Cunniffe N. J. Fedoroff N. V.. (2021). The persistent threat of emerging plant disease pandemics to global food security. Proc. Natl. Acad. Sci. 118:e2022239118. doi: 10.1073/pnas.2022239118, PMID: 34021073 PMC8201941

[ref102] Roberts R. Steenkamp E. T. Pietersen G. (2015). Three novel lineages of ‘Candidatus Liberibacter africanus’ associated with native rutaceous hosts of Trioza erytreae in South Africa. Int. J. Syst. Evol. Microbiol. 65, 723–731. doi: 10.1099/ijs.0.069864-0, PMID: 25395434

[ref57] Rodriguez C. I. Martiny J. B. H. (2020). Evolutionary relationships among bifidobacteria and their hosts and environments. BMC Genomics 21:26. doi: 10.1186/s12864-019-6435-1, PMID: 31914919 PMC6950798

[ref58] Rubio A. Pérez-Pulido A. J. (2021). Protein-coding genes of *Helicobacter pylori* predominantly present purifying selection though many membrane proteins suffer from selection pressure: a proposal to analyze bacterial pangenomes. Genes 12, 1–10. doi: 10.3390/genes12030377, PMID: 33800844 PMC7998743

[ref59] Seemann T. (2014). Prokka: rapid prokaryotic genome annotation. Bioinformatics 30, 2068–2069. doi: 10.1093/bioinformatics/btu153, PMID: 24642063

[ref60] Stamatakis A. (2014). RAxML version 8: a tool for phylogenetic analysis and post-analysis of large phylogenies. Bioinformatics 30, 1312–1313. doi: 10.1093/bioinformatics/btu033, PMID: 24451623 PMC3998144

[ref61] Stavrinides J. McCann H. C. Guttman D. S. (2008). Host-pathogen interplay and the evolution of bacterial effectors. Cell. Microbiol. 10, 285–292. doi: 10.1111/j.1462-5822.2007.01078.x, PMID: 18034865

[ref62] Steinert M. Ramming I. Bergmann S. (2020). Impact of Von Willebrand factor on bacterial pathogenesis. Front Med (Lausanne) 7:543. doi: 10.3389/fmed.2020.00543, PMID: 33015097 PMC7494747

[ref63] Strange R. N. Scott P. R. (2005). Plant disease: a threat to global food security. Annu. Rev. Phytopathol. 43, 83–116. doi: 10.1146/annurev.phyto.43.113004.133839 16078878

[ref103] Tannières M. Fowler S. V. Manaargadoo-Catin L. Lange C. Shaw R. M. (2020). First report of “Candidatus Liberibacter europaeus” in the United Kingdom. New Dis. Reps 41, 3. doi: 10.5197/j.2044-0588.2020.041.003, PMID: 34346758

[ref64] Tan Y. Wang C. Schneider T. Li H. de Souza R. F. Tang X.. (2021). Comparative Phylogenomic analysis reveals evolutionary genomic changes and novel toxin families in endophytic Liberibacter pathogens. Microbiol. Spectrum 9, e00509–e00521. doi: 10.1128/Spectrum.00509-21, PMID: 34523996 PMC8557891

[ref65] Teixeira D. C. Saillard C. Eveillard S. Danet J. L. Ayres A. J. Bové J. M. (2005). A new *Liberibacter* species, Candidatus *Liberibacter Americanus* sp. nov., Is Associated with Citrus Huanglongbing (Greening Disease) in São Paulo State, Brazil. International Organization of Citrus Virologists Conference Proceedings (1957–2010) 16.

[ref66] Tettelin H. Riley D. Cattuto C. Medini D. (2008). Comparative genomics: the bacterial pan-genome. Curr. Opin. Microbiol. 11, 472–477. doi: 10.1016/j.mib.2008.09.006 19086349

[ref67] Thapa S. P. De Francesco A. Trinh J. Gurung F. B. Pang Z. Vidalakis G.. (2020). Genome-wide analyses of *Liberibacter* species provides insights into evolution, phylogenetic relationships, and virulence factors. Mol. Plant Pathol. 21, 716–731. doi: 10.1111/mpp.12925, PMID: 32108417 PMC7170780

[ref68] Thompson S. Fletcher J. D. Ziebell H. Beard S. Panda P. Jorgensen N.. (2013). First report of ‘Candidatus *Liberibacter* europaeus’ associated with psyllid infested scotch broom. New Disease Rep 27:6. doi: 10.5197/j.2044-0588.2013.027.006

[ref69] Wamonje F. O. Zhou N. Bamrah R. Wist T. Prager S. M. (2022). Detection and identification of a ‘Candidatus *Liberibacter* solanacearum’ species from ash tree infesting psyllids. Phytopathology 112, 76–80. doi: 10.1094/PHYTO-02-21-0060-SC, PMID: 34346758

[ref70] Wang J. Haapalainen M. Schott T. Thompson S. M. Smith G. R. Nissinen A. I.. (2017). Genomic sequence of “Candidatus *Liberibacter* solanacearum” haplotype C and its comparison with haplotype a and B genomes. PLoS One 12:e0171531. doi: 10.1371/journal.pone.0171531, PMID: 28158295 PMC5291501

[ref71] Wang N. Pierson E. A. Setubal J. C. Xu J. Levy J. G. Zhang Y.. (2017). The Candidatus *Liberibacter*–host Interface: insights into pathogenesis mechanisms and disease control. Annu. Rev. Phytopathol. 55, 451–482. doi: 10.1146/annurev-phyto-080516-035513 28637377

[ref72] Yang Z. (1997). PAML: a program package for phylogenetic analysis by maximum likelihood. Bioinformatics 13, 555–556. doi: 10.1093/bioinformatics/13.5.555, PMID: 9367129

[ref73] Yang Z. (2007). PAML 4: phylogenetic analysis by maximum likelihood. Mol. Biol. Evol. 24, 1586–1591. doi: 10.1093/molbev/msm088, PMID: 17483113

[ref74] Yuan X. Chen C. Bassanezi R. B. Wu F. Feng Z. Shi D.. (2021). Region-wide comprehensive implementation of Roguing infected trees, tree replacement, and insecticide applications successfully controls Citrus Huanglongbing. Phytopathology 111, 1361–1368. doi: 10.1094/PHYTO-09-20-0436-R, PMID: 33356429

