# Supplementary material for: Comparative genomics of the Liberibacter genus reveals widespread diversity in genomic content and positive selection history
Source: Front Microbiol. 2023 Jun 26;14:1206094. doi: 10.3389/fmicb.2023.1206094 (PMC10330825; doi:10.3389/fmicb.2023.1206094)
Supplement: Supplementary file 3 [file Data_Sheet_1.PDF]

Supplementary Material

Comparative genomics of the Liberibacter genus reveals widespread diversity in genomic content and positive selection history.

Tiffany N. Batarseh, Sarah N. Batarseh, Abraham Morales-Cruz, Brandon S. Gaut*

*** Correspondence:** Brandon Gaut: bgaut@uci.edu

1 Supplementary Figures

1.1 Supplementary Figures

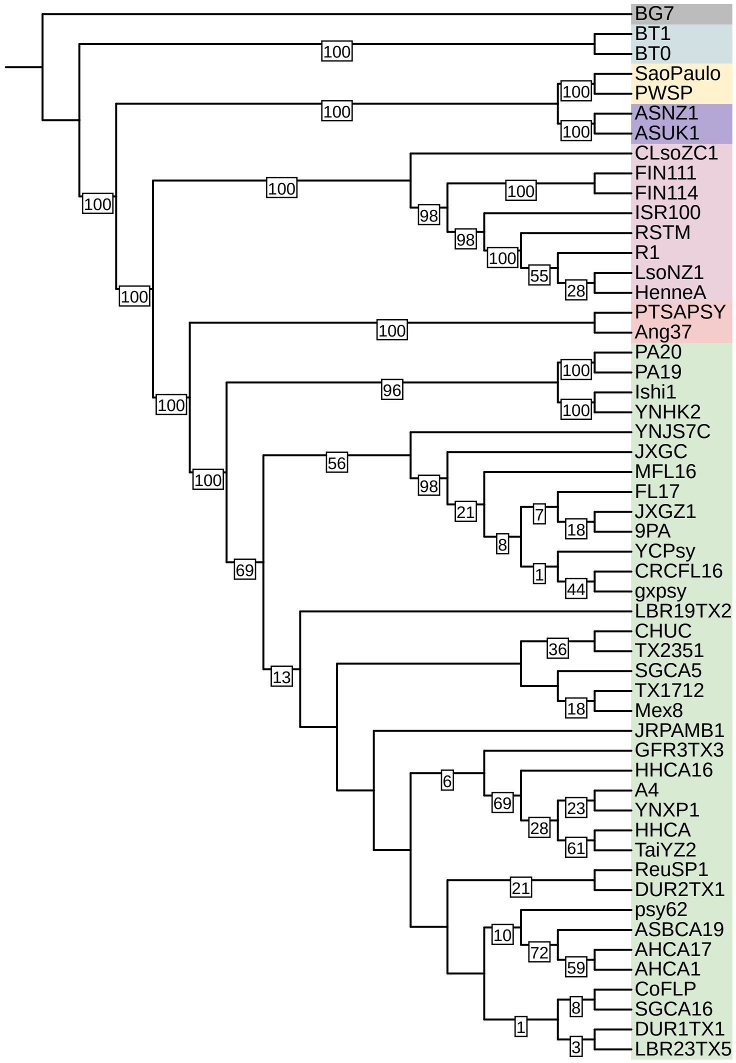

Supplementary Figure 1. The dendrogram depicting the phylogenetic relationships between *Liberibacter* accessions included in this study with bootstrap support denoted on the branches in boxes. The dendrogram was built using the nucleotide core gene alignment.

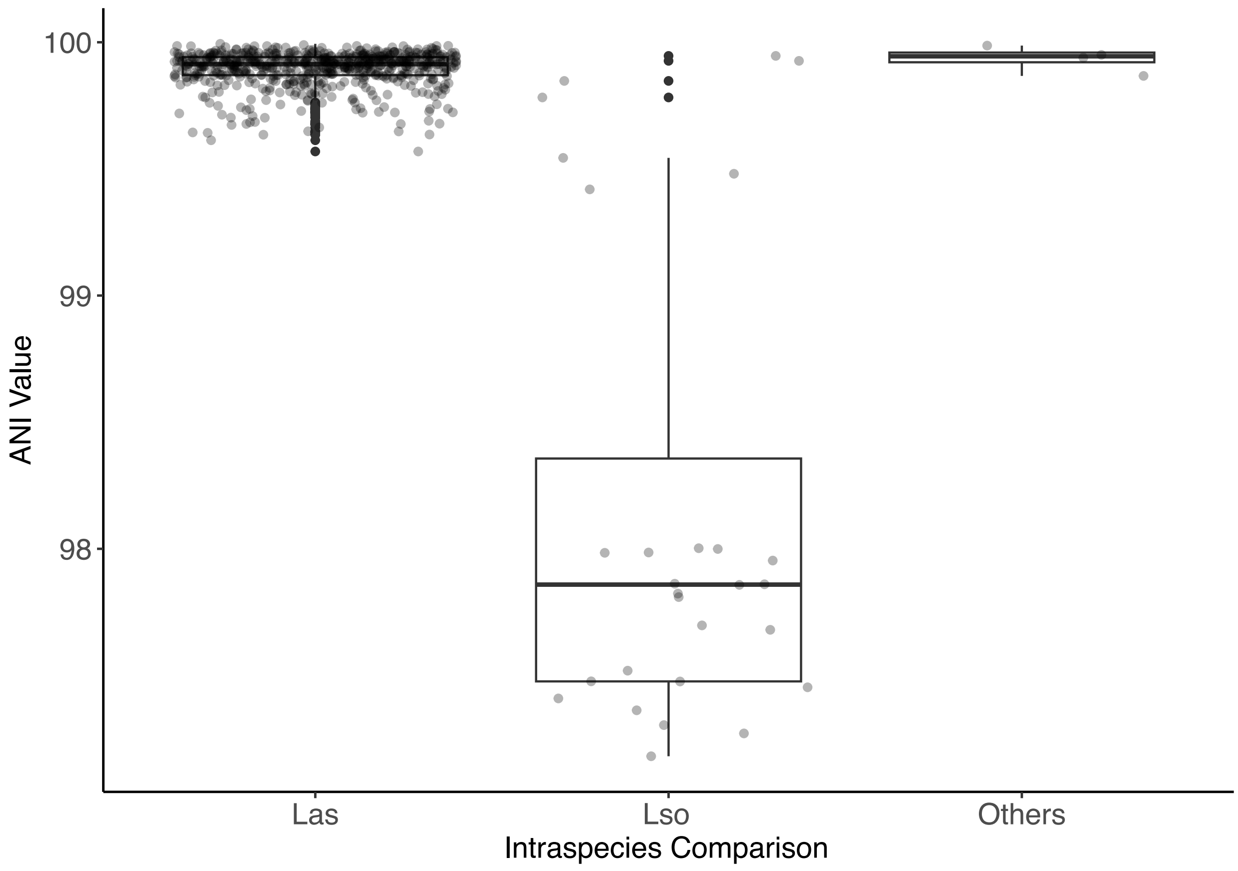

Supplementary Figure 2. Average nucleotide identity within the Las, Lso and all other species groups calculated with OrthoANI. The analysis fragmented the genome into 1,020 bp long fragments which were used for calculation of the percent identity.

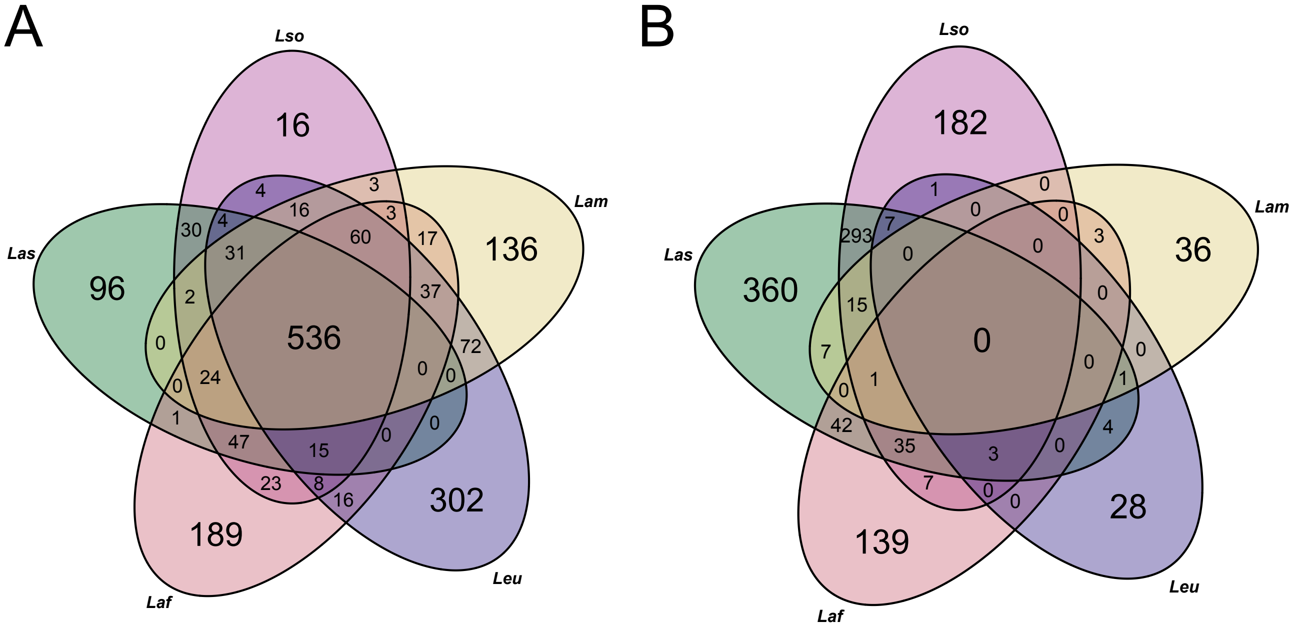

Supplementary Figure 3. Venn diagrams comparing number of the A) core and B) accessory gene (excluding singletons) between the 5 pathogenic species of *'Ca.* Liberibacter.' This analysis excluded *L. crescens* which is the reason for a slightly higher estimate of core genes (536 core genes across just the pathogenic species compared to 436 when *L. crescens* is included). The absolute counts of core and accessory genes per species are found in Table 2.

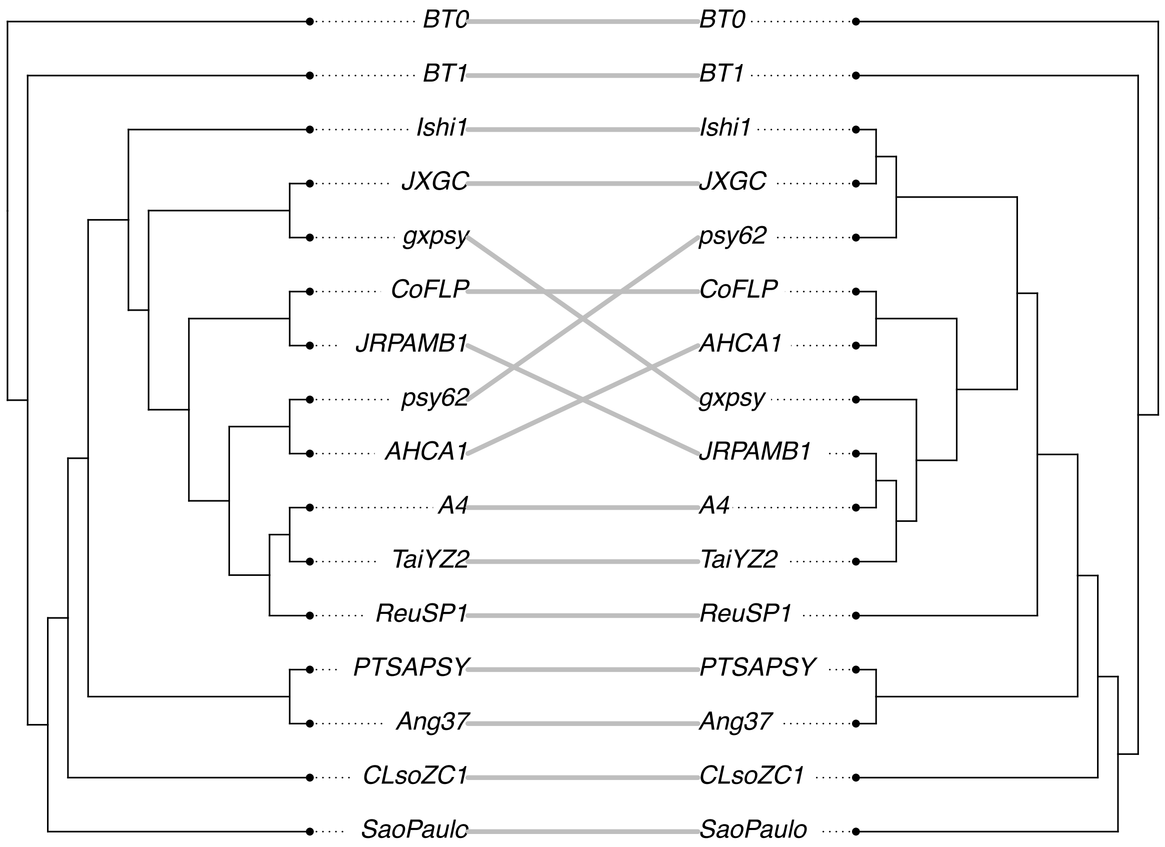

Supplementary Figure 4. A comparison of two likelihood trees representing the phylogenetic relationships of *Liberibacter* species based on different gene sequences. The tree on the left was built from the core gene alignment and the tree on the right was built from a whole genome alignment resulting from the Mauve analysis considering only the genome sequences that were chromosome level assemblies (= 1 contig).

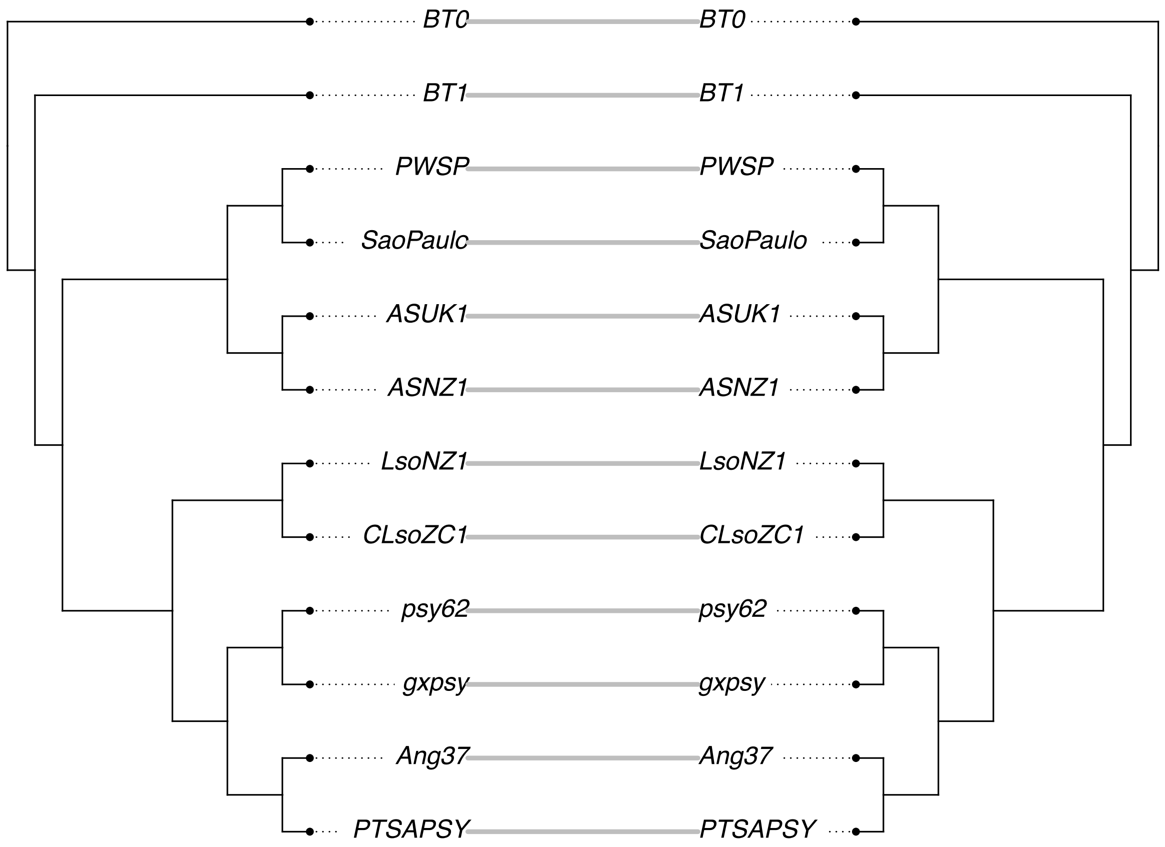

Supplementary Figure 5. A comparison of two likelihood trees representing the phylogenetic relationships of *Liberibacter* species based on different gene sequence alignments. The tree on the left was built from the core gene alignment and the tree on the right was built from a whole genome alignment resulting from the Mauve analysis which included a set of 12 genomes consisting of the two highest quality genomes available for all six Liberibacter species included in the analysis.
